# Tracking development assistance for health from China, 2007–2017

**DOI:** 10.1136/bmjgh-2019-001513

**Published:** 2019-10-08

**Authors:** Angela E Micah, Yingxi Zhao, Catherine S Chen, Bianca S. Zlavog, Golsum Tsakalos, Abigail Chapin, Stephen Gloyd, Jost Jonas, Paul H Lee, Shiwei Liu, Man Tat Alexander Ng, Michael R Phillips, Enrico Rubagotti, Kun Tang, Shenglan Tang, Mustafa Younis, Yunquan Zhang, Christopher J L Murray, Joseph L Dieleman

**Affiliations:** 1 Institute for Health Metrics and Evaluation, Seattle, Washington, USA; 2 Department of Global Health, University of Washington, Seattle, Washington, USA; 3 Ophthalmology, Medical Faculty Mannheim of the Ruprecht Karl University of Heidelberg, Mannheim, Germany; 4 School of Nursing, Hong Kong Polytechnic University, Hong Kong, China; 5 National Center for Chronic and Noncommunicable Disease Control and Prevention, Chinese Center for Disease Control and Prevention, Beijing, Hubei, China; 6 Tencent Healthcare, Shenzhen, Guangdong, China; 7 Shanghai Mental Health Center, School of Medicine, Shanghai Jiao Tong University, Shanghai, Shanghai, China; 8 Biotechnology, Universidad Regional Amazónica Ikiam, Ciudad de Tena, Napo, Ecuador; 9 Research Center for Public Health, Tsinghua University School of Medicine, Beijing, Hubei, China; 10 Duke Global Health Institute, Duke University, Durham, North Carolina, USA; 11 Department of Health Economics & Management, Jackson State University, Jackson, Mississippi, USA; 12 Hubei Province Key Laboratory of Occupational Hazard Identification and Control, School of Public Health, Wuhan University of Science and Technology, Wuhan, Hubei, China

**Keywords:** Health economics, Health policy, Health systems

## Abstract

**Introduction:**

In recent years, China has increased its international engagement in health. Nonetheless, the lack of data on contributions has limited efforts to examine contributions from China. Existing estimates that track development assistance for health (DAH) from China have relied primarily on one dataset. Furthermore, little is known about the disbursing agencies especially the multilaterals through which contributions are disbursed and how these are changing across time. In this study, we generated estimates of DAH from China from 2007 through 2017 and disaggregated those estimates by disbursing agency and health focus area.

**Methods:**

We identified the major government agencies providing DAH. To estimate DAH provided by each agency, we leveraged publicly available development assistance data in government agencies’ budgets and financial accounts, as well as revenue statements from key international development agencies such as the WHO. We reported trends in DAH from China, disaggregated contributions by disbursing bilateral and multilateral agencies, and compared DAH from China with other traditional donors. We also compared these estimates with existing estimates.

**Results:**

DAH provided by China grew dramatically, from US$323.1 million in 2007 to $652.3 million in 2017. During this period, 91.8% of DAH from China was disbursed through its bilateral agencies, including the Ministry of Commerce ($3.7 billion, 64.1%) and the National Health Commission ($917.1 million, 16.1%); the other 8.2% was disbursed through multilateral agencies including the WHO ($236.5 million, 4.1%) and the World Bank ($123.1 million, 2.2%). Relative to its level of economic development, China provided substantially more DAH than would be expected. However, relative to population size and government spending, China’s contributions are modest.

**Conclusion:**

In the current context of plateauing in the growth rate of DAH contributions, China has the potential to contribute to future global health financing, especially financing for health system strengthening.

**Key questions**

**What is already known?**

- There have been several scholarly efforts to estimate how much development assistance China has provided for health (DAH), primarily relying on information from AidData.
- Measuring DAH from China is difficult due to scarce statistics from government sources, lack of clear definition of foreign aid from China and distinction between aid commitment and disbursement.
- No study has yet been able to provide a time series of DAH from China that disaggregates contributions through bilateral and multilateral agencies for the past decade, which limits our understanding of the nature and evolution of China’s global health activities.

**What are the new findings?**

- Drawing on official government and international reports, we generated year-specific estimates of DAH from China including 4 major bilateral agencies and 10 multilateral agencies, and estimated that DAH from China reached US$652.3 million in 2017.
- Our estimate of DAH from China is different from yet comparable with the previous estimates that used different data sources and estimation approaches.
- China’s total DAH contribution was among the top 10 when ranked with the main traditional donor countries, although its disbursement relative to population size and government spending is small.

**What do the new findings imply?**

- China has become an emerging donor country for global health. This role has large potential to contribute to future global health financing.
- With consideration of the financing gaps for achieving the sustainable development goals, more detailed estimates of China’s and other emerging donors’ contributions to global health are needed to identify potential new sources of funding.

## Introduction

In the last three decades, China’s rapid economic growth has made it an important participant in the global economy. Beyond growing foreign trade and investment, China has also become an influential stakeholder in the global health landscape. In China’s efforts to link itself to Central and South Asia, the Gulf countries, North Africa and Europe—commonly referred to as the Belt and Road Initiative—global health is one of the important pillars.^1^ As part of this initiative, China has signed a memorandum of understanding with the WHO.^2^ In addition to these collaborations and events, China has provided resources to help contain the Ebola crisis in West Africa and engaged proactively with other donors such as the USA in establishing the Africa Centres for Disease Control and Prevention.^1 3 4^ Furthermore, China has started to provide more support to multilateral agencies. Besides WHO, China also began contributing to Gavi, the Vaccine Alliance in 2016.^5^ In 2018, China proposed its first development agency, the China International Development Cooperation Agency.^6–11^

These global health efforts have garnered a great deal of attention, largely because China’s approach to development assistance in general and for health in particular (DAH) is distinct from other traditional donors. The majority of China’s bilateral DAH disbursements are in kind. This means that health projects and services are usually directly delivered by China, avoiding direct cash transfer to recipients.^12^

Despite the attention and interest, scarce official statistics on contributions to development assistance by China has made estimating DAH contributions from China difficult and created uncertainty in terms of the scale and nature of DAH provided by China. Estimating DAH from China is difficult due to several reasons. First, what is considered ‘foreign assistance’ by the Chinese government is unclear despite the release of two government white papers on China’s foreign aid in 2011 and 2014.^13–16^ China’s definition of foreign assistance or development aid is different from the Western definition of official development assistance,^17^ and China also does not report to the Development Assistance Committee of the Organisation for Economic Co-operation and Development (OECD). This lack of clarity of definition limits efforts to examine its foreign assistance contributions. Second, there are challenges related to distinguishing between commitments and disbursements. Press releases on projects typically highlight the initially committed amounts for proposed projects and provide limited information on the actual amounts disbursed on the project. Third, there are concerns related to the accuracy of officially reported estimates of their foreign assistance, as with many of China’s official statistics—errors or statistical discrepancies may exist and users of official statistics should be cautious.^18^

Notwithstanding these challenges, there have been several studies that estimate DAH from China, although these studies have concluded with divergent results. Tang and colleagues estimated DAH from China from 2010 to 2013 using AidData and other sources, and estimated that the total DAH contributions from China was US$340 million in 2012 and $489 million in 2013,^1^ while Shajalal and colleagues estimated China contribution of DAH to Africa from 2000 to 2013 utilising AidData and concluded that in 2012 $978 million and in 2013 $487 million was contributed.^19^ Grepin and colleagues also estimated that a cumulative total of $3.0 billion was committed to Africa between 2000 and 2012^12^ for health, population, and water and sanitation projects. These previous studies rely on data from AidData’s Global Chinese Official Finance Dataset, which provides a compilation of China’s development spending based on news reports, government documents, recipient countries’ aid information systems, and scholarly and non-governmental organisations (NGOs) research. It is the only project-level database with disaggregated health sector information on DAH from China that is currently publicly available. However, this database primarily tracks bilateral contributions and does not include DAH contributions from China to the various multilateral entities.

Given China’s increasingly important role in the global economy, the current changes in commitments from traditional donors of DAH, and the gaps in financing required to achieve the Sustainable Development Goals, building a solid base of estimates of the contributions of emerging donors such as China is critical despite the paucity of data. We contribute to knowledge in this area by generating an estimate of DAH from China from 2007 through 2017 that is disaggregated by disbursing agency—bilateral and multilaterals—and health focus area and is based on methods and data different from those used previously. We also compare our estimates with previous estimates in order to broaden understanding of the range of estimates of DAH contributions from China.

## Methods

### Overview

DAH is the in-kind and financial resources transferred through international development agencies to low-income or middle-income countries for the purpose of maintaining or improving health.^20^ We defined DAH from China as DAH from the central government of China (People’s Republic of) through its own bilateral or other international development agencies. We did not include resources from the local government (provincial government and so on) of mainland China due to data availability; we also did not include private philanthropic contributions. To estimate the DAH provided by China, we leveraged official development assistance data reported in government agency budgets, financial accounts and yearbooks, as well as financial statements from key international development agencies such as the World Bank and the United Nations agencies. Our framework split DAH from China into two components—bilateral and multilateral contributions—and then aggregated the components to obtain the total amount of DAH. The bilateral component covered Chinese government agencies that provided DAH, while the multilateral component captured China’s contributions to multilateral entities such as United Nations agencies. Table 1 provides a list of agencies included and their data sources.

**Table 1:** List of bilateral and multilateral channels included

Channel	Source
Bilateral	
China National Health Commission (NHC)	Annual Department Final Account 2010–2017 Finance Yearbook of China 2007–2017
China Ministry of Commerce (MOFCOM)	Annual Department Final Account 2007–2017 White Paper on China’s Foreign Aid 2014 United Nations Office for the Coordination of Humanitarian Affairs (UNOCHA) Financial Tracking Service 2014–2015
China Ministry of Education (MOE)	Annual Department Final Account 2007–2017 Education Yearbook of China 2007–2014 Ministry of Education Website 2015–2017
China Export-Import Bank (EXIM)	Almanack of China’s Finance and Banking 2007–2008, 2010–2014 AidData 2000–2014
Multilateral	
World Health Organization (WHO)	WHO Programmatic and Financial Report 2007–2017
United Nations Population Fund (UNFPA)	UNFPA Report on contributions by member states and others to UNFPA and revenue Projections 2007–2017 UNFPA Statistical and Financial review—Audited Financial report 2007–2017
Joint United Nations Programme on HIV/AIDS (UNAIDS)	Joint United Nations Programme on HIV/AIDS Financial Report and Audited Financial Statement 2007–2017
United Nations Children’s Fund (UNICEF)	UNICEF Financial Report and Audited Financial Statement—Executive Board documents 2007–2017
World Bank	World Bank International Development Association 16th–19th Replenishment Report World Bank International Development Association Financial Statement 2007–2017 World Bank project-level health disbursement through correspondence 2007–2017
Regional Development Banks	Asian Development Bank project-level health disbursement through correspondence 2007–2017 Asian Development Fund 6th–12th replenishment reports Asian Development Bank Annual Report 2007–2017 African Development Bank project-level health disbursements through correspondence 2007–2017 African Development Fund 9th–14th replenishment reports Inter-American Development Bank Annual Financial Statements 2007–2016
The Global Fund to Fight AIDS, Tuberculosis and Malaria (GFATM)	Global Fund Grants in detail and Disbursements database 2007–2017 Global Fund Annual Report 2007–2017 Global Fund Pledges & Contributions Report 2007–2017
Gavi, the Vaccine Alliance	Gavi Annual financial reports 2007–2017

### Estimating DAH from China through bilateral agencies

A concern regarding China’s quantification of official development assistance (ODA) is related to its classification of loans at market rate. Our definition of DAH from China is aligned with our previous definition of DAH as the ‘in-kind and financial resources transferred through international development agencies to low-income or middle-income countries for the purpose of maintaining or improving health’. To align our estimates with this definition, for each relevant government entity, we evaluated whether we could identify the in-kind or financial resources disbursed that was targeted at recipients in low-income or middle-income countries and that was for the primary purposes of improving or maintaining health. We also evaluated the terms of the resources disbursed. We excluded any organisation that disbursed loans at market rates.

We identified four Chinese government agencies that are the primary agencies responsible for the disbursement of DAH. The identification of these agencies was informed by a white paper on foreign aid published by the Chinese State Council in 2011 and other research.^13 15 21^ The four agencies were the National Health Commission (NHC), the Ministry of Commerce (MOFCOM), the Ministry of Education (MOE) and the Export-Import Bank of China (EXIM). We excluded other government entities from the analyses based on six reasons: (1) The Ministry of Finance and the People’s Bank of China did not disburse development aid bilaterally, mostly contributing to multilateral agencies and the South-South Cooperation Fund has not yet disbursed funds. (2) The China Development Bank, Silk Road Fund and China-Africa Development Fund disbursed loans, but the loans were not concessional and therefore not considered as DAH. (3) The Red Cross Society of China mostly disbursed aid in emergency situations, and our definition of DAH precludes emergency response humanitarian support; and the limited number of non-emergency projects are usually through Ministry of Commerce. (4) The Ministry of Foreign Affairs, Ministry of Human Resources and Social Security, National Development and Reform Commission, All China Woman Federation, Ministry of Science and Technology, Ministry of Agriculture, Ministry of Civil Affairs, and State Oceanic Administration had no health-related aid disbursements based on literature review. (5) For non-governmental organisations including the China Foundation for Poverty Alleviation and Lifeline Express, the disbursement data available were incomplete and not disaggregated. (6) The Chinese embassies and consulates and the Chinese Centre for Disease Control and Prevention also disbursed DAH, although the disbursements were administratively reported under other government agencies that we included —the Ministry of Commerce and the National Health Commission. eTable 2 in the online supplementary file provides additional details on the basis of inclusion or exclusion of each government agency in the analysis.

10.1136/bmjgh-2019-001513.supp1 Supplementary data

We also calculated administrative expenses for all the bilateral channels. We defined administrative expenses as the costs associated with administering grants and loans. We used the administrative cost ratio of MOFCOM as a proxy for all the bilateral agencies, and generated the administrative cost ratio estimate by dividing the basic expenditure for MOFCOM’s foreign affairs over project expenditure for foreign affairs reported in the annual department account. We used the year-specific ratio for 2007–2017.

#### National Health Commission (NHC)

NHC manages international medical teams deployed abroad and implements other public health programmes abroad. To estimate the amount of DAH disbursed by NHC, we extracted (1) the amount of development assistance disbursed from the NHC’s Department Final Account, available for 2010–2017,^22^ and (2) the overall Chinese development assistance expenditure from the central government, from the Finance Yearbook of China, available for 2007–2017.^23^ We calculated that, on average, 3.0% of development assistance was disbursed through NHC based on the 2010–2017 data. We multiplied this amount by the total amount of development assistance for the years where the NHC data were unavailable to estimate development assistance for NHC for 2007–2009. We assumed that all development assistance from the NHC is health related.^24^

#### Ministry of Commerce (MOFCOM)

MOFCOM is the central ministry in China’s foreign aid disbursement. Development assistance provided through MOFCOM includes complete aid projects (infrastructure turn-key projects) such as hospital construction, material aid projects such as drug and medical equipment donations, as well as short training programmes for public health professionals. Development assistance programmes provided through MOFCOM are either grants or interest-free loans.^15^ To estimate MOFCOM’s DAH contributions, we generated MOFCOM’s development assistance total, extracted from MOFCOM’s Department Final Account 2007–2017.^25^

We assumed that 13.8% of MOFCOM’s development assistance is for health. This estimate was based on data from the 2014 white paper on China’s foreign aid that reports that 80 of 580 complete aid projects between 2010 and 2012 were hospital projects.^14^

Additionally, we used project-level data on Ebola funding from the United Nations Office for the Coordination of Humanitarian Affairs (UNOCHA) Financial Tracking Service website to supplement our data based on the support that China provided during the Ebola crisis,^26^ which was channelled through MOFCOM.

#### Ministry of Education (MOE)

MOE provides scholarships for foreign students to study in China. We considered all scholarships provided to foreign medical and health sciences students as DAH. To generate DAH through MOE, we estimated the cost per foreign medical student on government scholarship and multiplied it by the number of foreign medical students on scholarship.

To estimate the cost per medical science student, we extracted the number of incoming foreign students on Chinese government scholarship from the Education Yearbook of China (2007–2014)^27^ and the MOE website (2015–2017).^28 29^ Using these data and the MOE development assistance total extracted from the MOE’s Department Final Account (2007–2017),^30^ we calculated the cost of government scholarship per person per year. Given that medical and health sciences students typically cost more than other students in terms of tuition, we adjusted for the difference in costs based on a 2015 Ministry of Finance notice.^31^ We estimated that medical and health sciences students cost 10% more than other students based on additional data provided in this notice that listed the standard tuition and stipend fees for medical, science and art students.

To estimate the total number of foreign medical students on scholarship, we extracted the number of incoming medical students on a Chinese government scholarship (2008, 2009, 2011) and the number of all incoming students on a Chinese government scholarship from the Education Yearbook of China.^27^ Based on these data, we calculated the percentage of medical sciences students among all students to be 11.14%. We assumed that 11.14% of all incoming students are medical students, and we used this number to generate the number of medical students on a foreign scholarship for the years with missing data.

#### Export-Import Bank (EXIM)

EXIM Bank is the only policy bank in China that provides concessional loans. We extracted the concessional loan disbursements from the Almanack of China’s Finance and Banking (2007–2008, 2010–2014).^32^ To determine the share of loans that were health related, we calculated the share of total EXIM loan disbursements that were health related for each year, as reported in the AidData database.^16^ We obtained an average estimate of 1.5%. We generated estimates for the remaining missing years of data using linear regression that estimated the year-over-year DAH growth rate, and the growth rate was used to generate DAH disbursement for 2015–2017.

### Estimating DAH from China through multilateral organisations and public–private partnerships

We used data from IHME’s Financing Global Health 2018 Development Assistance for Health database to estimate DAH contributions from China to multilateral aid agencies.^33^ We identified the following agencies as having received contributions from China: the United Nations Population Fund, the Joint United Nations Programme on HIV/AIDS, the United Nations Children’s Fund, WHO, the World Bank, the African Development Bank, the Asian Development Bank, the Inter-American Development Bank, the Global Fund and Gavi. This particular study did not include China’s contribution to NGOs. For the World Bank, where we do not have income data for China, we generated contribution estimates using replenishment data. We split the 3-year replenishment amount over the 3 years of the 16th to 19th replenishments to obtain China’s contributions to the World Bank from 2008 to 2017.^34^ We validated our use of replenishment data by extracting China’s and other countries’ contributions to the World Bank International Development Association (IDA) 16th, 17th and 18th replenishments and comparing the number with the contribution we extracted from the OECD Creditor’s Reporting System. The trend of contributions from major donors was similar, and we therefore used China’s replenishment contribution as a proxy of the contribution to World Bank IDA. We further disaggregated the DAH contribution to World Bank IDA from China by multiplying China’s contributing proportion over World Bank IDA’s total health envelope. For the remaining disbursing agencies, a more detailed description of the original methodology used to obtain the estimates in the database can be found elsewhere^20^ and in the online supplementary file.

### Aggregating China’s total DAH contribution

For the four bilateral channels, we converted the volume in Chinese Renminbi (CNY) into US dollars (USD) based on year-specific exchange rates extracted from the OECD exchange rate database. We deflated disbursements to constant 2018 USD using the International Monetary Fund deflator series. We aggregated China’s total DAH contribution by adding up the bilateral and multilateral contributions.

### Estimating the health focus areas of DAH from China

We also estimated DAH from China by health focus area. Health focus areas of interest include HIV/AIDS, malaria, tuberculosis, reproductive and maternal health, newborn and child health, other infectious diseases, non-communicable diseases, health system strengthening and ‘other’.^20 35^ We disaggregated the multilateral channels using the same methodology as in Chang and colleagues.^20^ Due to a lack of project-level information for the bilateral channels, we were only able to allocate the health focus area on the basis of available literature and other information included in the Department Final Accounts. We allocated the National Health Commission’s DAH to health system strengthening and ‘other’ on the basis of the amount of ‘medical aid’ (which was footnoted as ‘medical team provision’) and ‘other types of aid’, listed in the Department Final Account.^22^ We allocated the Ministry of Commerce’s and Export-Import Bank’s DAH to health system strengthening, considering that their projects, as described in the Commerce Yearbook, are health infrastructure, equipment and medical products provision.^36^ We also allocated the Ministry of Education’s DAH to health system strengthening because the scholarships for medical and health sciences students support human resources for health.^27^

### Comparing DAH from China with other traditional donor countries

We compared DAH from China with other traditional donor countries that are members of the OECD Development Assistance Committee to highlight the global context for DAH contributions. The definition of DAH captures both in-kind and financial flow of resources, focuses on the specific channels used to disburse resources, the primary intent of the funds to improve or maintain health and that the recipients are low-income or middle-income countries. In this analysis, we include in-kind contributions, grants, interest-free and concessional loans and exclude loans disbursed at market rates. These characteristics are the criteria that are used in this analysis to define the boundary of DAH for China and hence make them comparable to those generated for the other donors.

We extracted DAH contributions from 23 traditional donor countries from IHME’s Financing Global Health 2018 Development Assistance for Health database.^33^ We extracted the population size, gross domestic product and total government spending data from IHME’s Financing Global Health and the Global Burden of Disease databases and calculated per person contribution and per government spending for comparison. We calculated the percentage of DAH flows to health system strengthening using health focus area information, and we summed health system strengthening and all disease-specific health system strengthening (HSS) (HIV/AIDS-HSS, malaria-HSS, tuberculosis-HSS, reproductive and maternal health-HSS, newborn and child health-HSS, and so on) spending to generate our numerator of health system strengthening.

We used a simple ordinary least squares regression to examine donor DAH contributions conditional on national income. The fitted values provided the average expected DAH contribution for donors given their level of economic development and enabled an assessment of observed donor contributions relative to what could be expected given their level of economic development. We also compared our estimates of DAH from China with previous estimates from Tang and colleagues,^1^ Shajalal and colleagues^19^ and AidData.^16^ All analysis was completed using Stata V.13. We reported DAH contributions in constant 2018 USD. A detailed description of the methodology used can be found in the online supplementary file.

### Patient and public involvement statement

We did not involve patients or the public in our work.

## Results

**Figure 1:**
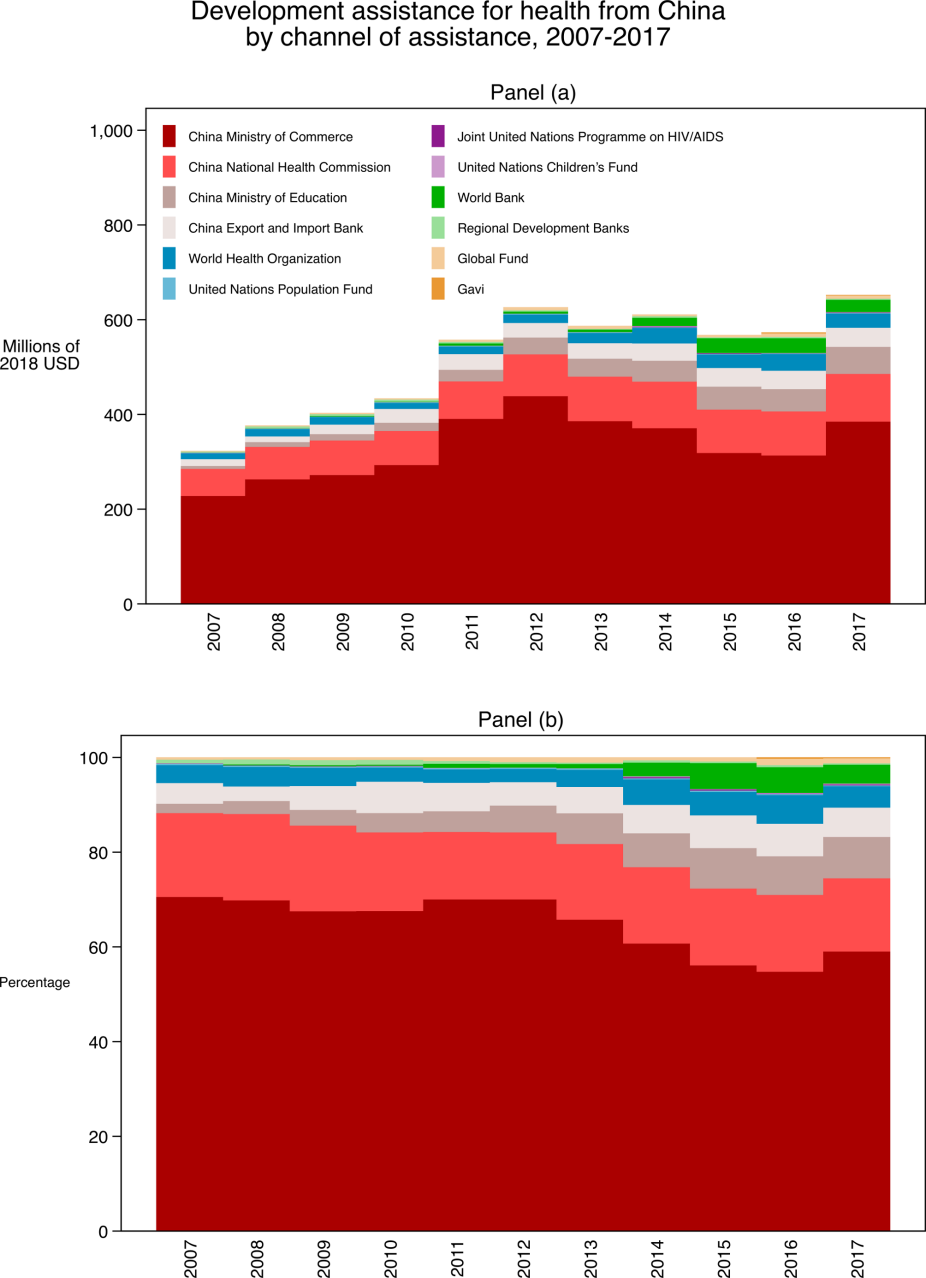
Development assistance for health from China by disbursing agency. Note: Development assistance for health from China by (a) disbursing agency in values in 2018 US dollars, (b) and in relative percentage each year. Regional development banks include the African Development Bank, Asian Development Bank and the Inter-American Development Bank.

Figure 1 illustrates the DAH provided by China, disaggregated by disbursing agency. Panel (a) highlights the amount of DAH provided by China over time. Between 2007 and 2017, China’s development assistance for health contributions increased from $323.1 million to $652.3 million. Panel (b) shows the annual share of contribution from disbursing agencies across time. The majority of DAH from China is disbursed through its bilateral agencies. MOFCOM disbursed at least 50% of DAH from China over the entire period, although this share has been decreasing in recent years. NHC also disbursed a large share, 15.4% in 2017. This proportion has been steady over time. WHO is the largest multilateral disbursing agency of DAH from China and disbursed 4.5% of DAH from China in 2017.

**Figure 2:**
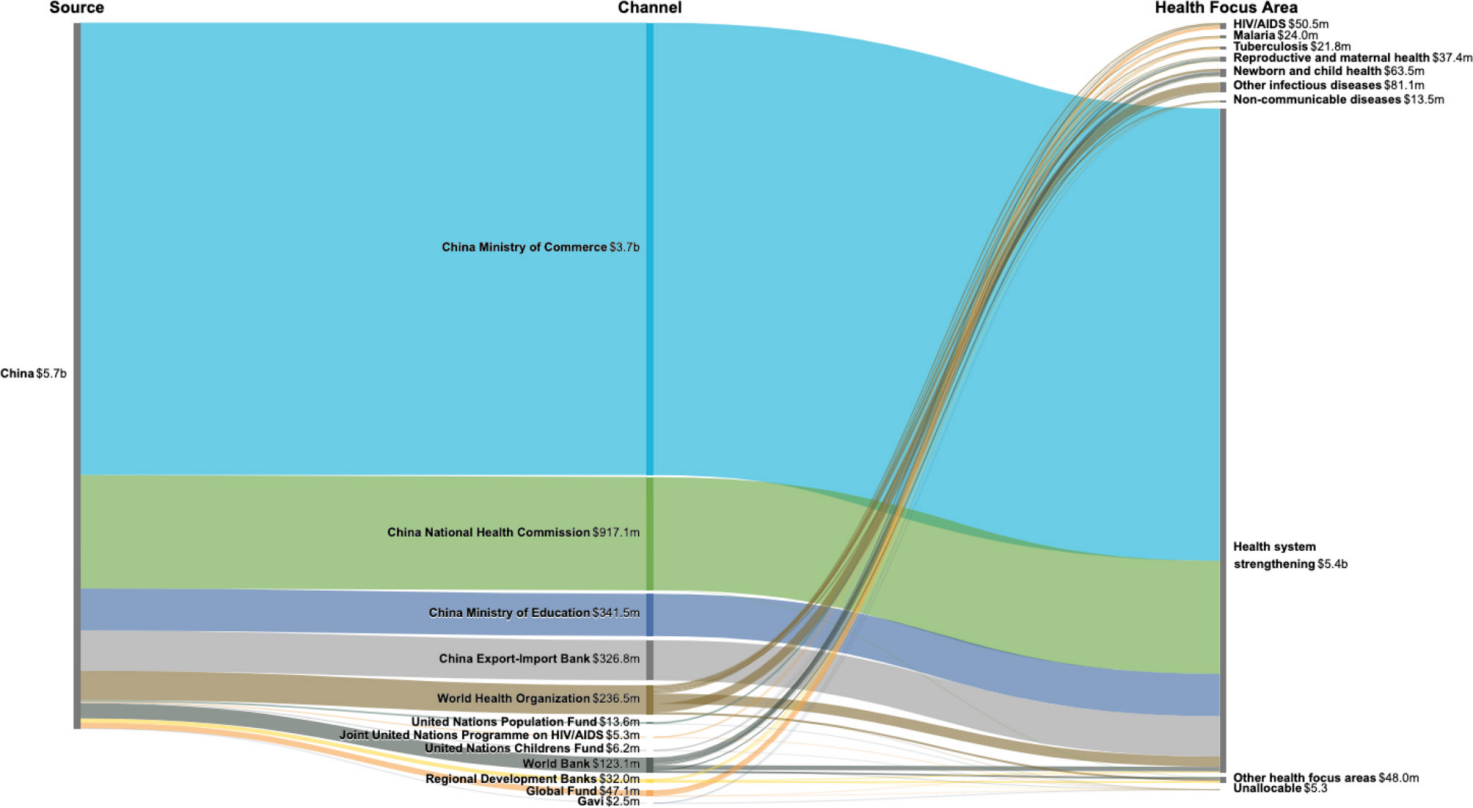
Flows of development assistance for health from China, disbursing agency and health focus area, 2007–2017. Note: Values are aggregate disbursement for 2007–2017, in 2018 US dollars. Regional development banks include the African Development Bank, Asian Development Bank and the Inter-American Development Bank. Other health focus areas refer to development assistance for health for which we have project-level information that is not identified as funding for the health focus areas tracked. Unallocable refers to development assistance for health we do not have project-level information and health focus area cannot be determined.

Figure 2 highlights the flow of funds through disbursing agencies and health focus areas from 2007 through 2017. Over the past 11 years, $5.7 billion in DAH was provided by China, and MOFCOM disbursed $3659.7 million, 64.1% of all DAH from China; NHC disbursed $917.1 million, 16.1% of all DAH from China; and MOE and EXIM disbursed $341.5 (6.0%) and $326.8 (5.7%) million, respectively. The rest was disbursed through multilateral channels, which constitutes only 8.2% of all DAH disbursed by China. Among the multilateral channels, the WHO ($236.8 million, 4.1%) and the World Bank ($123.1 million, 2.2%) were the major disbursing agencies. Health system strengthening ($5.4 billion, 94.1%) is the main area of focus of DAH from China, while other infectious diseases ($81.1 million, 1.4%) and newborn and child health ($63.5 million, 1.1%) are the other ranked health focus areas of DAH from China.

Table 2 compares total DAH disbursement from China and other traditional donor countries. It also compares the total amount relative to the population, government spending and the share that targeted health system strengthening. Between 2015 and 2017, China provided more DAH than that provided by half of the donor countries that are members of the OECD’s development assistance committee and was among the top 10 highest contributors. The DAH contribution measured relative to the population size highlights the burden of providing assistance on the citizens of the contributing country, while the DAH contribution measured relative to overall government spending is a proxy for the level of generosity exhibited. When considering DAH provided per person and per government spending, the citizens of China provided $0.4 per person towards DAH, about 1% when compared with the USA and UK. Relative to China’s government budget, China provided $158.5 per $1 million total government spending.

**Table 2:** Comparing development assistance for health from China with major sources, 2015–2017

Source	Total DAH (millions)	DAH per capita	DAH per US$1 million government spending	Proportion of DAH flows to health system strengthening	Gross domestic products per capita
USA	(*1*) 13 479	(*5*) 41.77	(*5*) 1896	(*18*) 27%	(*10*) 60 187
UK	(*2*) 3494	(*4*) 53.48	(*1*) 2622	(*20*) 26%	(*17*) 49 856
Germany	(*3*) 1437	(*14*) 17.54	(*12*) 723	(*7*) 36%	(*14*) 54 933
Canada	(*4*) 1022	(*10*) 28.14	(*8*) 1140	(*16*) 28%	(*9*) 61 087
Japan	(*5*) 1004	(*17*) 8.00	(*18*) 358	(*11*) 31%	(*12*) 57 890
France	(*6*) 973	(*15*) 15.00	(*16*) 520	(*19*) 26%	(*16*) 50 708
Netherlands	(*7*) 709	(*6*) 41.40	(*6*) 1503	(*24*) 18%	(*8*) 61 953
Norway	(*8*) 677	(*2*) 128.94	(*3*) 2238	(*17*) 27%	(*2*) 116 825
Sweden	(*9*) 659	(*3*) 66.76	(*4*) 1903	(*21*) 25%	(*7*) 71 024
China	(*10*) 598	(*24*) 0.44	(*23*) 159	(*1*) 92%	(*24*) 8700
Australia	(*11*) 441	(*13*) 18.29	(*14*) 629	(*6*) 41%	(*5*) 78 245
Italy	(*12*) 334	(*20*) 5.53	(*20*) 268	(*9*) 35%	(*19*) 41 417
Belgium	(*13*) 287	(*11*) 25.24	(*11*) 863	(*3*) 48%	(*15*) 54 784
South Korea	(*14*) 279	(*19*) 5.54	(*13*) 680	(*2*) 63%	(*21*) 30 425
Switzerland	(*15*) 262	(*8*) 31.25	(*10*) 913	(*14*) 30%	(*3*) 101 437
Denmark	(*16*) 224	(*7*) 39.16	(*9*) 1007	(*15*) 30%	(*6*) 72 021
Spain	(*17*) 184	(*21*) 3.96	(*21*) 248	(*8*) 36%	(*20*) 37 188
Ireland	(*18*) 143	(*9*) 30.70	(*7*) 1313	(*23*) 24%	(*4*) 83 260
Finland	(*19*) 102	(*12*) 18.48	(*15*) 592	(*12*) 31%	(*13*) 55 693
Luxembourg	(*20*) 78	(*1*) 134.77	(*2*) 2400	(*22*) 25%	(*1*) 134 757
Austria	(*21*) 77	(*16*) 8.88	(*19*) 300	(*4*) 46%	(*11*) 58 046
New Zealand	(*22*) 34	(*18*) 7.38	(*17*) 477	(*13*) 31%	(*18*) 46 580
Portugal	(*23*) 31	(*22*) 3.00	(*22*) 243	(*10*) 33%	(*23*) 26 429
Greece	(*24*) 13	(*23*) 1.22	(*24*) 88	(*5*) 44%	(*22*) 26 742

Note: Values are 2015–2017 average values expressed in 2018 USD. The colours in the figure reflect the relative values of the various measures on a pro rata basis. For each column, the darkest green represents the highest values for that column, while the darkest red represents the lowest values. The rank is provided in parenthesis. The health system strengthening includes sector-wide approach/health system strengthening, HIV/AIDS-health system strengthening, malaria-health system strengthening, tuberculosis health system strengthening, reproductive and maternal health-health system strengthening, newborn and child health-health system strengthening, other infectious diseases-health system strengthening and non-communicable diseases-health system strengthening.

DAH, development assistance for health.

It is worth noting that the percentage of health aid that targeted health system strengthening is significantly larger for China than for other donors. Over the 3-year period, China disbursed 91.8% of its health aid to health system strengthening, while in comparison, most countries disbursed less than 40% of their DAH to health system strengthening. The USA and UK targeted 26.5% and 25.7%, respectively, of all DAH flows to health system strengthening.

**Figure 3:**
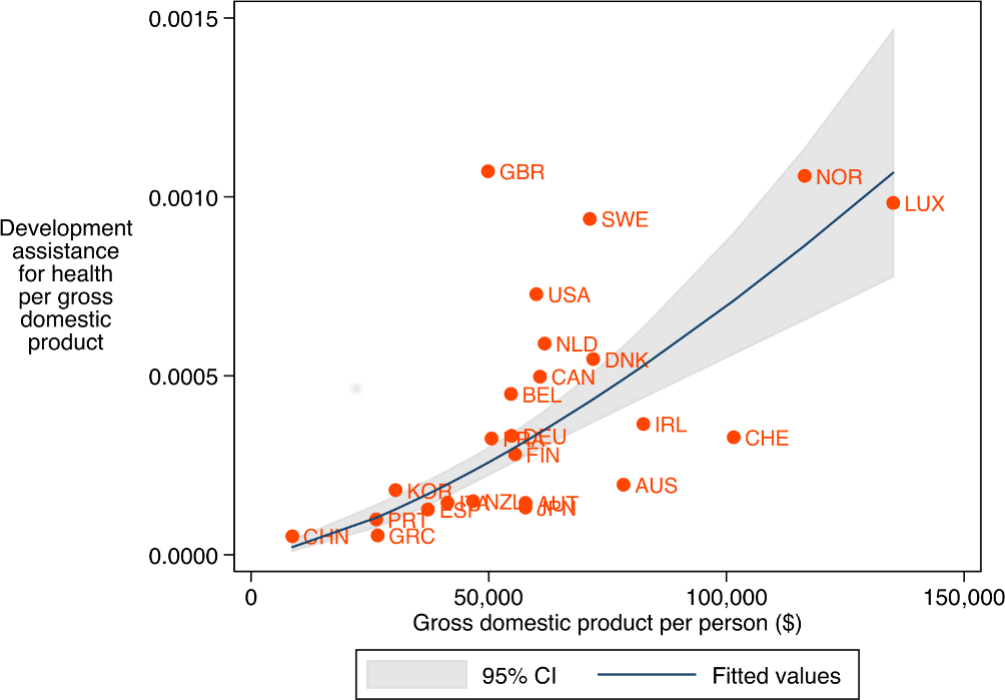
Expected and observed development assistance for health (DAH) contribution as a share of gross domestic product (GDP) conditional on gross domestic product per person of donor country. Note: Blue line represents the expected DAH contribution as a share of gross domestic product given a country’s income level. Solid orange dots represent the observed DAH contributions as a share of GDP. Grey area highlights the 95% CI. Placement above or below blue indicates DAH contributions above or below expected levels given level of economic development. Y-axis shows DAH contributions as a share of GDP. X-axis shows lagged GDP per person. Both variables are logged in the regression analysis. AUT, Austria; AUS, Australia; BEL, Belgium; CAN, Canada; CHE, Switzerland; CHN, China; DNK, Denmark; ESP, Spain; FIN, Finland; FRA, France; GBR, United Kingdom; GRC, Greece; IRL, Ireland; ITA, Italy; JPN, Japan; KOR, South Korea; LUX, Luxembourg; NLD, Netherlands; NOR, Norway; NZL, New Zealand; PRT, Portugal; SWE, Sweden; USA, United States. CI, Confidence Interval

Figure 3 shows the average DAH disbursement rate and the observed disbursement rate of the donor countries conditional on level of economic development. Countries below the fitted line contributed less DAH than expected relative to their level of development. Although China is among the countries at the lower end of economic development, DAH contributions from China are greater than expected. At the other extreme, we have countries such as Norway, which has a high level of economic development and also contributes more than is expected given its level of development.

Table 3 compares our estimate of DAH with previous estimates from Tang and colleagues,^1^ Shajalal and colleagues,^19^ as well as the raw data on health commitments from AidData.^16^ Comparing estimates for 2012 and 2013, the years with data in all four studies, our estimates were $626 and $587 million, while the estimates from Tang and colleagues were $382 and $549 million, estimates from Shajalal and colleagues were $1098 and $547 million, estimates from AidData were $150 and $286 million. In both years, our estimates are higher than the estimates reported by Tang and colleagues. In 2012, both our estimate and Tang and colleagues’ estimate is lower than that reported by Shajalal and colleagues. The reported estimate is more than double Tang’s estimate and a third more than our estimate. In 2013, our estimate of DAH is the highest among the studies.

**Table 3:** Comparing estimates of development assistance for health from China, in millions of 2018 US dollars

Year	IHME	Tang and colleagues	Shajalal and colleagues	AidData
2007	323		576	202
2008	377		195	256
2009	403		1109	201
2010	434	525	1024	101
2011	558	390	496	287
2012	626	382	1098	150
2013	587	549	547	286
2014	611			297
2015	568			
2016	573			
2017	652			

Note: Values are expressed in 2018 USD. Estimates for Tang and colleagues and Shajalal and colleagues are extracted from the tables and figures, deflated to 2018 USD from 2011 USD. The estimate for Shajalal and colleagues include water and sanitation projects and is restricted to projects in Africa only. Estimates for AidData are extracted from its Global Chinese Official Finance Dataset 2000–2014 database through extracting the projects tagged ‘health’ in sector name.

## Discussion

This study estimated DAH from China from 2007 to 2017, leveraging official development assistance data from government sources and financial statements from key international development agencies. This study is important in that it is the most-updated one to quantify DAH from China for over a decade, and to distinguish between bilateral and multilateral contributions. The data generated allow us to examine the trends in China’s global health activities and compare contributions from China through its various disbursing agencies.

Our estimate of DAH from China is different yet comparable with the previous estimates from Tang and colleagues^1^ and Shajalal and colleagues.^19^ The three estimates used different data sources and estimation approaches. Our different estimate when compared with Tang and colleagues is expected as our inclusion of DAH through multilaterals are captured by contribution and replenishment data, while Tang and colleagues used data on contribution and share of development banks. Additionally, differences in how estimates for bilateral contributions are generated may explain some differences. Tang and colleagues generated their estimates based on the number of projects and their average value and used the mean project value to fill in missing values. The much higher estimate of DAH obtained by Shajalal and colleagues in 2012 may be related to how they defined DAH. Their estimate is inclusive of water and sanitation projects, while the other two studies do not include allied sectors for health. Also, the estimate of Shajalal and colleagues is restricted to Africa, whereas our estimates and those from Tang and colleagues did not have recipient country information. According to the 2011 white paper issued by the government of China, about 45.7% of overall official development assistance contributions from China flows to Africa.^13^ Another source of difference could be that our estimates are based on disbursements instead of commitments to account for differences between what a country initially commits to an international agency and what actually gets disbursed in that specific year. While comparable to previous estimates, the novel set of estimates reported here are critical for gaining confidence in the amount and nature of DAH from China. Using alternative data and methods strengthens the characterisation of China as an emerging donor for the health sector.

Beginning in the 2000s, China has become a prominent stakeholder in the global health arena. This increased engagement is reflected in its contributions to DAH, which have increased significantly over this period. This trend is corroborated in the government white paper on foreign aid released in 2011, which reports that China’s financial resources for foreign aid have increased rapidly, averaging 29.4% from 2004 to 2009.^13^ China has sustained large current account surpluses and has increased its foreign exchange holdings to more than 1.5 trillion USD, which has allowed it to expand its foreign aid and investment plans.^37^

Furthermore, China’s enhanced economic and trade engagement with Africa, beginning in the 2000s, has facilitated its increased engagement in the global development and health space. At each of the Forum on China-Africa Cooperation meetings organised every 3 years, since 2006, in either Beijing or an African country, large aid and investment commitments have been made. At the September 2018 meeting, an additional 50 medical aid projects were committed by the Chinese government in continuous support of the Africa Centres for Disease Control and Prevention and the China-Africa friendship hospital system.^38^

While China made increased bilateral commitments to global health in Africa, Southeast Asia and Latin America, it should be emphasised that bilateral DAH from China is different from that of other traditional donors. China usually provides in-kind DAH where the health projects and services are delivered directly by China.^12^ These aid delivery approaches avoid cash transfers and recipient government structural systems as such, funds may usually not be transferred locally in the recipient countries.

Besides the increase in China’s bilateral DAH contributions, China has also increased its contributions to multilateral organisations and public–private partnerships. China has historically supported UN agencies, especially WHO. In 2016, DAH from China disbursed through WHO reached $34.7 million, nearly half of all multilateral contributions from China. As a multilateral institution, WHO receives the largest share of China’s multilateral DAH contributions for several reasons, including that WHO is the first international organisation that has had a Chinese-national director-general.^1^ More recently, China has started to contribute to the World Bank and in 2008 participated in the IDA replenishment round.^39^ In 2014, when concessional loans were accepted as contributions in the IDA replenishment round, China’s contribution to the World Bank saw nearly a threefold increase to $17.7 million. Nonetheless, compared with its huge bilateral aid disbursements and the capital base of its newly committed development funds (China-Africa Development Fund, South-South Cooperation Fund, Silk Road Fund) and banks (Asian Infrastructure Investment Banks, New Development Bank),^1^ it still remains uncertain whether China will continue to emphasise contributions to existing multilateral mechanisms.

China’s total amount of DAH has surpassed that of some members of the OECD Development Assistance Committee and was among the top 10 when ranked with main traditional donor countries. Also, relative to its level of economic development, DAH contributions from China are significant, although relative to its population size and level of government spending, contributions are modest. China’s pledges, despite being welcomed by developing countries and the international development community, have been widely criticised by domestic citizens.^40^ Critics have challenged that China is still a developing country itself with a large number of people living in poverty and that the government should prioritise domestic social welfare rather than delivering aid to foreigners.^40^ The government has indicated that China’s foreign aid is ‘win-win’ and ‘could open up the Africa market’ to ease domestic concerns.^41^

Additionally, DAH from China seems to emphasise health system strengthening more through providing and training health workers, constructing health infrastructure and providing drugs and other medical products. Globally, in 2017 only 28.7% of health financing flowed to health system strengthening and sector-wide approaches^20^; more emphasis has been placed on prevention, diagnosis and treatment in vertical programmes. China also provided DAH towards maternal and child health and other infectious diseases, although these make up a relatively smaller share of contributions. Nonetheless, it is also possible that China’s lesser focus on vertical programmes is due to its comparatively weak capacity in delivering disease-specific aid. China, despite its huge success in decreasing maternal and child mortality and trying to eliminate malaria, has relatively limited experience in operating these programmes overseas. Therefore, there has been some scepticism over whether the approach in China works in other, more decentralised countries, where government capacity and structure can be weak.^4^

While China has increased DAH disbursement dramatically for the past decades, it remains a recipient of DAH. China received a total of $802.3 million in assistance from the Global Fund from 2003 to 2013, and in 2014 graduated from the Global Fund’s support.^42^ In 2016, a total of $244.5 million in assistance was given to China through major bilateral donors including Germany, USA, and UK, and multilateral agencies including the World Bank and Asian Development Bank, and the Bill & Melinda Gates Foundation.^35^ Nonetheless, the relative volume of DAH received by China is lower than DAH contributions from China.

Although our tracking of China’s health aid is advantageous for several reasons related to scope, it is not without limitations. To start with, we rely heavily on government sources as our primary source to estimate the bilateral envelope of DAH from China. These sources, despite being official, are inconsistent in some years and require caution in analysing.^43^ For instance, we observed in 1 year that the foreign assistance disbursed through the Ministry of Commerce was slightly larger than the foreign assistance disbursed through the central government, the latter of which should be the sum of disbursements from all government ministries. Also, we did not find other sources of information regarding the share of Ministry of Commerce’s total aid that is for health besides the white paper on foreign aid, and thus we used the information on project numbers from the white paper as a constant proportion. The same challenge applies to the share of Export-Import Bank’s total loan that is for health where we used the health proportion calculated based on data from AidData. We are aware that the proportion of health projects should not be fixed and is likely to increase over time. This uncertainty could influence our final estimate, for example, if the Ministry of Commerce’s health aid proportion was 10% or 20% instead of 13.8%, our total envelope of DAH from China would range between $546.3 and $825.3 million in 2017 instead of $652.3 million. Due to lack of data availability, we are not able to produce a more accurate estimate of the proportion. Nonetheless, we believe that the yearbook, final accounts and reports are the best available information we could extract and help us understand the total aggregate of the financial portfolio for health. We also include in the online supplementary file several sensitivity analysis scenarios to examine the robustness of our results.

Second, due to data availability, we are not yet able to track development assistance disbursed through NGOs, the military, and the Central Committee of the Communist Party of China, or at the local government level, or the health-related emergency aid from the Red Cross Society of China. China has only a few NGOs operating abroad, including the China Foundation for Poverty Alleviation. We were unable to disaggregate the health share of their annual expenditures listed in their publicly available annual report. However, according to the few available financial reports, we observed that DAH disbursed through this channel is less than 1% of the total DAH disbursed.^44^ Similarly, we were not able to disaggregate the health-related emergency aid from the Red Cross since the majority of its aid projects were for emergency settings such as in flood zones and after earthquakes, and the available reported data were not disaggregated. Their department account only listed development aid in 2013 and 2016, and DAH disbursed through this channel is less than 0.1% of the total DAH disbursed.^45^ Furthermore, although we did not include DAH disbursed through the Communist Party of China and the military due to data availability, we noted that the International Liaison Department of the Communist Party of China and the People’s Liberation Army Navy provided some development aid projects related to health, including the hospital ship ‘Peace Ark’.^46 47^ Our DAH estimates are also restricted to aid disbursed through the central government level, thus excluding the disbursements for medical teams and a few other health aid projects that are implemented by local governments, especially provincial governments. Typically, the medical teams are funded by three sources—central National Health Commission, provincial National Health Commission and provincial hospitals.^48^ Due to significantly diverse salary and stipend data provided by the hospitals, we are unable to track this proportion. For the local government level, we compared the total foreign aid disbursed by the central government and total foreign aid disbursed by the local government using information from the finance yearbooks. We found that the local government only started disbursements after 2010, and this was estimated to be at most 0.77% of the central government aid budget.^23^ Therefore, we restricted our estimates to central government disbursements. These exclusions may result in an underestimation of contributions of DAH from China.

Without project-level data on bilateral disbursements, we were also unable to provide a more detailed disaggregation of the health focus areas in terms of programme areas and recipient information. Our allocation of health focus areas is largely based on literature reviews and could lead to misclassification. For example, we allocated all MOFCOM DAH to health system strengthening based on literature guidance that its aid projects usually entail hospital and other facility construction and drug and equipment donations. We are aware that drug donations include some malaria treatment drugs but were unable to disaggregate that out to malaria.

## Conclusion

China is an emerging donor country for global health. Its DAH contribution has dramatically increased over the past decade, reaching a total $652.3 million in 2017 and ranking in the top 10 among traditional donors. While the country’s DAH is relatively small compared with its government budget, its DAH heavily focuses on health system strengthening. While globally only about 28.7% of financial resources flows to health system strengthening efforts, China’s emerging role has the potential to contribute to future global health financing. Additionally, as China’s influence in the global health landscape widens, an official centralised database that publishes more details on the development projects financed by China will be essential to global health resource tracking efforts. Such information is not only critical to understanding China’s contributing to global health financing but also for recipient country level and regional level cooperation between donors.
