# Supplementary material for: Tracking development assistance for health from China, 2007–2017
Source: BMJ Glob Health. 2019 Oct 8;4(5):e001513. doi: 10.1136/bmjgh-2019-001513 (PMC6782043; doi:10.1136/bmjgh-2019-001513)
Supplement: Supplementary data [file bmjgh-2019-001513supp001.pdf]

SUPPLEMENTARY METHODS ANNEX

Tracking development assistance for health from China, 2007-2017

Corresponding: Angela E. Micah, PhD
amicah@uw.edu
+01-206-897-3816
Institute for Health Metrics and Evaluation
2301 5th Ave, Suite 600
Seattle, WA 98121

Version: Aug 9, 2019

Table of Contents

SUPPLEMENTARY METHODS ANNEX 1

Tracking development assistance for health from China, 2007-2017 1

Part 1. Introduction 3

Part 2. Estimating DAH from China through bilateral agencies 4

Tracking development assistance for health from the National Health Commission (NHC) 7

Tracking development assistance for health from the Ministry of Commerce (MOFCOM) 9

Tracking development assistance for health from the Ministry of Education (MOE) 11

Tracking development assistance for health from the Export-Import Bank (EXIM) 14

Calculating the administrative cost contribution for China's bilateral agencies 21

Part 3. Estimating DAH from China Through Multilateral Organizations and Public-Private Partnerships 22

Tracking development assistance for health from the World Bank 23

Part 4. Aggregating China's total DAH contribution and disaggregating the health focus area 26

Estimating the health focus area of DAH from China 26

Part 5. Comparing DAH from China with other traditional donor countries and previous estimates 27

Part 6. Comparing DAH with total official development assistance 28

Part 7. Sensitivity analysis for China's development assistance for health 30

Reference 36

Part 1. Introduction

This methods appendix provides detailed information on how we generated the development assistance for health (DAH) contributions from China over the period 2007 through 2017.

DAH is defined as the in-kind and financial resources transferred to low-income and middle-income countries for the purpose of maintaining or improving health. DAH from China is thus defined as DAH from the central government of China (People's Republic of) through its own bilateral or other international agencies. We did not include resources from the local government (provincial government, etc.) of mainland China, due to data availability; resources from Hong Kong, Macau or Taiwan; and also exclude private philanthropic contributions.

To estimate the DAH provided by China, we split DAH from China into two components – bilateral and multilateral contributions – and then aggregated the components to obtain the total amount of DAH. The bilateral component referred to the Chinese government agencies that provided DAH bilaterally, and we estimated the amount through these government agencies' department account, yearbooks and other sources; the multilateral component captured China's contributions to multilateral entities such as United Nations agencies, and we generated estimates based on the financial statements and annual reports of the multilateral entities.

Subsequent sections of this methods annex provides information on the specific data sources, interpolation and regression strategies used to generate the estimates. Part 2 provides details on the methodology for estimating DAH contributed through bilateral entities. Part 3 describes the methodology used to estimate DAH from China contributed through the multilateral organizations and public-private partnerships. Part 4 highlights the process of aggregating the two components of DAH contributions described in the preceding sections and further details how we disaggregated DAH contributions into relevant focus areas of health. Part 5 presents additional details on the comparative analysis of DAH contributions from China and other traditional donors and the ordinary least squares regression analysis. Part 6 compares official development assistance and development assistance for health from major donors in 2010-2016. Part 7 presents the additional sensitivity analysis of our estimates.

Part 2. Estimating DAH from China through bilateral agencies

In order to estimate DAH from China's bilateral entities, we first conducted a comprehensive literature search to identify potential government agencies involved in official development assistance work and screened them for any health related components. We used the 2011 White Paper on Foreign Aid published by the Chinese State Council (1), and previous research on China's development assistance to determine the specific government entities included in the analysis. eTable 1 details the sources of information used to generate the list of government agencies included in the analysis. All government agencies considered for inclusion with explanation for their inclusion or exclusion are summarized in eTable 2.

eTable 1 Data sources reviewed in landscaping analysis used to generate list of potential government agencies involved in development assistance for health.

Type	Literature author and title	Reference
Government report	The State Council. China's Foreign Aid (2011)	(1)
Government report	The State Council. China's Foreign Aid (2014)	(2)
Journal article	Kitano, Naohiro, and Yukinori Harada. "Estimating China's foreign aid 2001–2013."	(3)
Research report	JICA Research Institute. A Note on Estimating China's Foreign Aid Using New Data: 2015 Preliminary Figures.	(4)
Journal article	Shajalal, Mohon, et al. "China's engagement with development assistance for health in Africa."	(5)
Journal article	Liu P, Guo Y, Qian X, Tang S, Li Z, Chen L. China's distinctive engagement in global health.	(6)
Journal article	Bräutigam, Deborah. "Aid 'With Chinese Characteristics': Chinese Foreign Aid and Development Finance Meet the OECD-DAC Aid Regime."	(7)
Journal article	Tang, Kun, et al. "China's Silk Road and global health."	(8)
Book	Brautigam D. *The Dragon's Gift: The Real Story of China in Africa*.	(9)
Research report	Mckinsey & Company. Dance of the lions and dragons: How are Africa and China engaging, and how will the partnership evolve?	(10)
Journal article	Yanzhong, Huang. "Domestic politics and China's health aid to Africa."	(11)
Journal article	Dreher, Axel, and Andreas Fuchs. "Rogue aid? The determinants of China's aid allocation."	(12)

eTable 2 Listing of potential government agencies providing DAH reviewed and explanation for inclusion or exclusion in analysis

Agency	Inclusion	Note
Export-Import Bank of China	Yes	The key policy bank disbursing concessional loans, including health related loans (13).
Ministry of Commerce	Yes	Central and largest agency disburing development aid prior to the establishment of the new aid agency in 2018 (14,15).
Ministry of Education	Yes	The line ministry managing education related aid, including scholarships for medical science students (16).
National Health Commission	Yes	The line ministry managing health related aid (17).
China Center for Disease Control	No	Although the China Center for Disease Control (CDC) disburses aid itself on occasion , the department account of the National Health Commission (NHC) listed the income and disbursement of CDC administratively in its department final account. Thus, we did not include CDC seperately as a disbursing agency (18).
International Development Cooperation Agency	No	New agency established in 2018 (19).
All China Woman Federation	No	Based upon official website search and literature search, we determined that it has no heath related disbursement (20).
China-Africa Development Fund	No	The fund is part of the China Development Bank, and its funding is given at commercial market rates and thus is also not considered as foreign aid by the Chinese government (21,22).
China Development Bank	No	The bank is not a policy bank, and its overseas loans are at commercial market rates (23).
Chinese embassies and consulates	No	Aid disbursements of the embassies and consulates, especially the Economic and Commercial Counselor's Offices are already administratively included in the Ministry of Commerce account (15).
Ministry of Agriculture	No	Based upon official website search and literature search, we determined that it has no heath related disbursement (24).
Ministry of Civil Affairs	No	Based upon official website search and literature search, we determined that it has no heath related disbursement (25).
Ministry of Finance	No	Besides providing funding to each ministry for development aid, it does not disburse development aid bilaterally. It mostly contributes to multilateral agencies (e.g. directly to the World Bank, and to the International Planned Parenthood Federation through the China Family Planning Commission) (26).
Ministry of Foreign Affairs	No	Based upon official website search and literature search, we determined that it hs no heath related disbursement (1).
Ministry of Human Resource and Social Security	No	This Ministry is involved in medical team related aid, but according to its website and literature review, its involvement is primarily administrative. It does not directly disburse health related aid (27).
Ministry of Science and Technology	No	Based upon official website search and literature search, we determined that it has no heath related disbursement (28).

National Development and Reform Commission	No	Based upon official website search and literature search, we determined that it has no heath related disbursement (29).
Non-governmental organizations	No	We identified the non-governmental organizations participating in overseas health work through screening entities listed in the China Foundation Center database (30). Four were identified: China Foundation for Poverty Alleviation, the Amity Foundation, Lifeline Express and Yunnan Foundation of Folk Friendship. However, their website did not provide disaggregated DAH, and we failed to get a response through email follow up requests. The total volume of these agencies is estimated to be less than 1% of our total DAH estimates (31).
People's Bank of China	No	This agency is responsible for multilateral contribution, i.e. provide funding to the African Development Bank and Inter-American Development Bank, but does not disburse aid bilaterally (32).
Red Cross Society of China	No	The Red Cross mostly disbursed aid in emergency situations, and our definition of DAH precludes non-health emergency response humanitarian support. Available data did not allow disaggregation of health and non-health disbursement. According to its own department final account (33), the total aid volume is estimated to be less than 0.1% of total DAH.
Silk Road Fund	No	Based upon official website search and literature search, we determined that it has no health related disbursement (34).
South-South Cooperation Fund	No	The fund was established with an initial contribution of 2 billion USD in 2015 to support the sustainable development goals however it had not yet disbursed any funds by 2017 (35).
State Oceanic Administration	No	Based upon official website search and literature search, we determined that it has no heath related disbursement (36).

After reviewing, four agencies were determined to be included in our bilateral envelope: the National Health Commission, the Ministry of Commerce, the Ministry of Education, and the Export-Import Bank of China.

Tracking development assistance for health from the National Health Commission (NHC)

The National Health Commission manages international medical teams deployed abroad and implements other public health programs, including maternal and child health programs, in foreign countries. We assumed that all of National Health Commission's development aid is DAH (17).

Step 1: Extracting all National Health Commission foreign aid volume

We extracted the development assistance disbursement of National Health Commission (formerly Ministry of Health from 2010-2012, National Health and Family Planning Commission from 2013-2017) from the Department Final Account of Ministry of Health/National Health and Family Planning Commission from 2010-2017 (18). The disbursement amount is reported in 10,000 CNY. At this step, we are missing the following years of data: 2007-2009.

Step 2: Extracting China's government disbursement for foreign aid

We extracted the total amount of foreign aid disbursed by the government from the Finance Yearbook of China 2007-2016 (37). Using this source, we are able to extract data on government foreign aid disbursement from 2007 to 2016.

Step 3: Generating estimate of National Health Commission health aid volume

Based on step 2 and 3, we have data on foreign aid disbursed through the National Health Commission and the government's overall envelope. We calculated the annual share of total aid disbursed through the National Health Commission for the years with overlapping data – 2010 through 2016. We calculated the average share of disbursement through the National Health Commission during 2010-2016 to be 0.030323. We assume this to be the share of total aid disbursed through the National Health Commission and used this estimated constant proportion to estimate the National Health Commission share of total aid for all the years – 2007 through 2009 – for which we had data on the total aid envelope. In essence, from 2007-2009, we estimated that 3.03% of all Chinese government foreign aid disbursements flowed through the National Health Commission.

eTable 3 reports the raw data and the results from the methods discussed above.

eTable 3 Estimate generation for development assistance for health from the National Health Commission

Year	Foreign aid extracted from Department Account	Foreign aid budget extracted from the Department Account	Central government overall disbursement for foreign aid extracted from the Finance Yearbook	Share of disbursement through NHC	Estimated DAH through NHC
2007			11154	0.030323	338.2205
2008			12559	0.030323	380.8241
2009			13296	0.030323	403.172
2010	401.0519		13611	0.029465	401.0519
2011	425.5492		15898	0.026768	425.5492
2012	473.8635		16691	0.02839	473.8635
2013	509.3817		17049	0.029878	509.3817
2014	539.5631		18457	0.029234	539.5631
2015	510.0696	610	19387	0.02631	510.0696
2016	557.3066	610	15660	0.035588	557.3066
2017	623.3547	580	16870	0.036951	623.3547

Note: Aid volume is expressed in 10,000 CNY.

eFigure 1 Estimate generation for development assistance for health from the National Health Commission

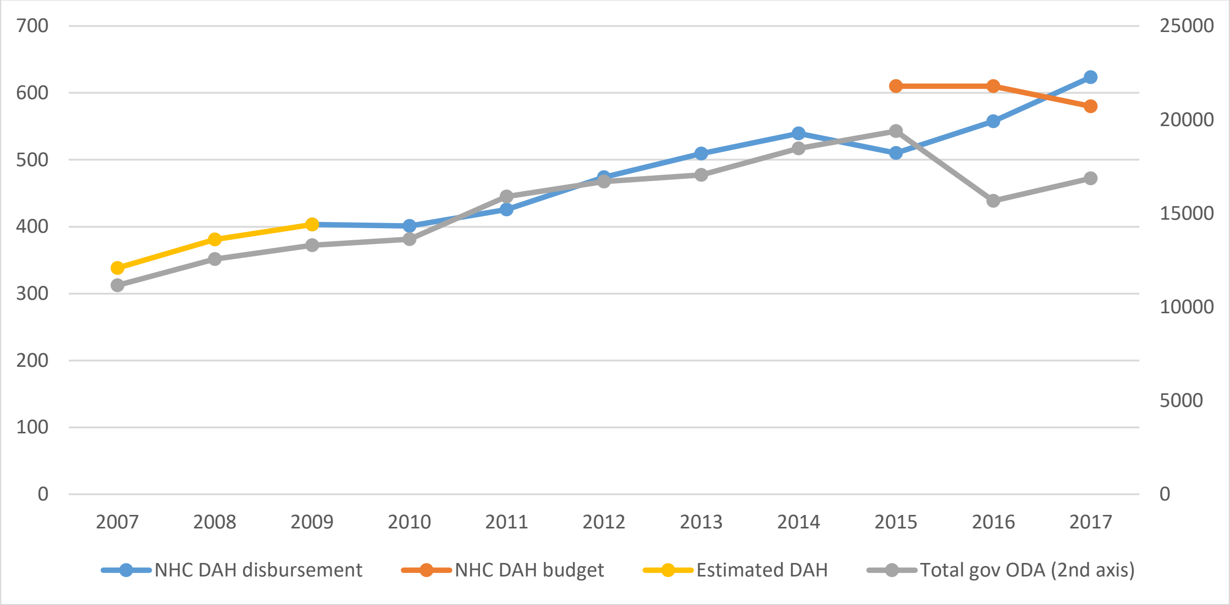

Note: Aid volume is expressed in 10,000 CNY.

Tracking development assistance for health from the Ministry of Commerce (MOFCOM)

The Ministry of Commerce is the central ministry in China's foreign aid disbursement. Development assistance provided through the Ministry of Commerce includes complete aid projects (infrastructure turn-key projects) such as hospital construction, material aid projects such as drug and medical equipment donations, as well as short training programs for public health professionals in collaboration with the National Health Commission (14).

Step 1: Extracting all Ministry of Commerce foreign aid volume

We extracted the development assistance disbursement of the Ministry of Commerce from the Department Final Account of Ministry of Commerce from 2007-2017 (15). The disbursement amount is reported in 10,000 CNY.

Step 2: Generating estimate of Ministry of Commerce health aid disbursement

We extracted from the White Paper on China's Foreign Aid (2014) that "out of all 580 complete aid projects between 2010 and 2012, 80 were hospital projects" (2). We used this proportion, 0.137931 as a proxy for the health aid percentage of all Ministry of Commerce aid. We then get the estimated health aid from the Ministry of Commerce 2007-2017 by multiplying the Ministry of Commerce foreign aid disbursement with 0.137931.

Step 3: Supplementing funding to Ebola

Additionally, we also utilized project level data on Ebola funding from the United Nations Office for the Coordination of Humanitarian Affairs (UNOCHA) Financial Tracking Service website (38) to supplement our data based on the support that China provided during the Ebola crisis, which was channeled through the Ministry of Commerce. For a more detailed explanation and example of supplement funding to Ebola, please refer to Page 31 of IHME's Financing Global Health 2017 Supplementary Methods Annex at
http://www.healthdata.org/sites/default/files/files/policy_report/FGH/2018/IHME_FGH2017_Methods_Annex.pdf

eTable 4 reports the raw data and the results from the methods discussed above.

eTable 4 Estimate generation for development assistance for health from the Ministry of Commerce

Year	Foreign aid extracted from Department Account	Estimated DAH through MOFCOM
2007	9767.904	1347.297
2008	10558.25	1456.31
2009	10887.23	1501.687
2010	11839.37	1633.016
2011	15177.99	2093.515
2012	17013.96	2346.752
2013	15206.13	2097.396
2014	14202.96	1959.029
2015	12810.12	1766.913
2016	13623.41	1879.09

2017	17274.15	2382.641

Note: Aid volume is expressed in 10,000 CNY.

eFigure 2 Trends in observed and estimated development assistance for health from the Ministry of Commerce

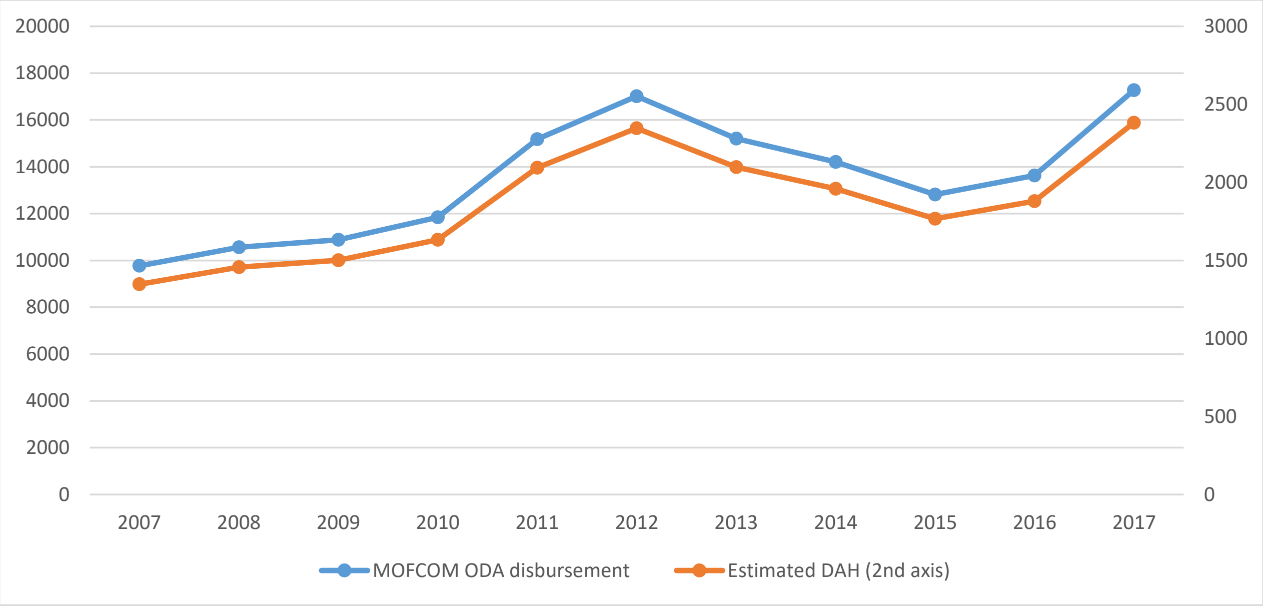

Note: Aid volume is expressed in 10,000 CNY.

eFigure 3 Trends of observed and estimated MOFCOM official development assistance, total government aid, gross domestic product and export volume

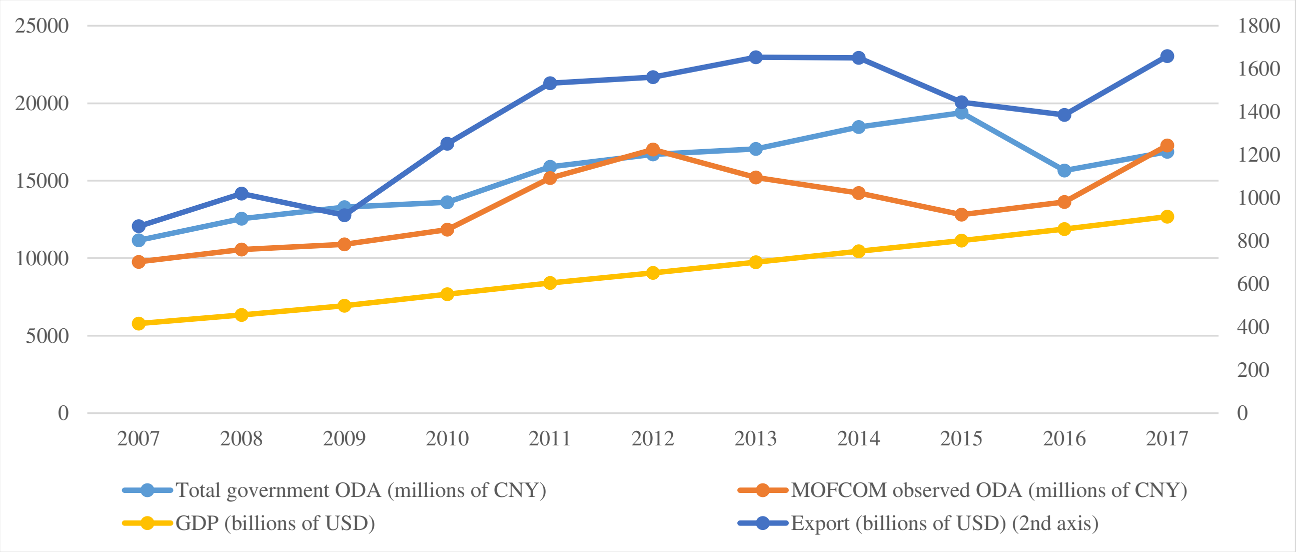

Tracking development assistance for health from the Ministry of Education (MOE)

The Ministry of Education provides scholarships for foreign students to study in China (16). We considered all scholarship provided to foreign medical and health sciences students as DAH.

Step 1: Extracting all MOE foreign aid volume

We extracted the incoming student scholarship disbursement of Ministry of Education from the Department Final Account of Ministry of Education from 2007-2017 (39). The disbursement amount is reported in 10,000 CNY.

Step 2: Estimating cost of Chinese government scholarship per person

We extracted the number of incoming foreign student on Chinese government scholarship from the Education Yearbook of China 1994-2014 (16) and the MOE website 2015-2016 (40,41). We selected the 2007-2016 number of incoming foreign student on scholarship, and the MOE foreign aid volume 2007-2016, and calculated the cost of government scholarship per person per year. We then calculated the overall average cost of scholarship by averaging the cost from 2007-2016. We used this number as our cost for Chinese government scholarship per person for that specific year. We calculated the three-year average cost for year 2006 using cost of 2007, 2008 and 2009, and held this cost constant for years before 2006.

Given that medical and health sciences students typically cost more than other students in terms of tuition, we adjusted for the difference in costs based on a 2015 Ministry of Finance notice (42). We estimated that medical and health sciences students cost 10% more than other students based on additional data provided in this notice that listed the standard tuition and stipend fee for medical students, science students and art students.

eBox 1 Process for estimating additional cost for medical and health sciences students

We extracted a 2015 Ministry of Finance notice on the scholarship amount for different categories and discipline students.

Category	Discipline	Tuition	Accommodation	Living stipend	Insurance	Total
Undergraduate	1	20000	8400	30000	800	59200
	2	23000	8400	30000	800	62000
	3	27000	8400	30000	800	66200
Master student	1	25000	8400	36000	800	70200
	2	29000	8400	36000	800	74200
	3	34000	8400	36000	800	79200
Doctoral student	1	33000	12000	42000	800	87800
	2	38000	12000	42000	800	92800
	3	45000	12000	42000	800	99800

Number are in CNY per year.
Discipline 1: Philosophy, economy, law, education, literature, history, management;
Discipline 2: Science, engineer, agricultural
Discipline 3: Art, medical and health science

We estimated the average cost of a student in discipline 3 among three categories (undergraduate, master and doctoral), (81733), and similarly for discipline 1 and 2 (74366), and arrived at the conclusion that medical students on average cost 10% more.

Step 3: Estimating number of medical students on Chinese government scholarship

We extracted number of incoming medical students on Chinese government scholarship from the Education Yearbook of China for year 2008, 2009 and 2011. We selected the respective 2008, 2009 and 2011 number of all incoming student on

Chinese government scholarship and calculated the average percentage of medical student among all students. We obtained an estimate of 0.111427, and we used this number as our constant proportion for medical students among all students from 2007, 2010 and 2012-2016. At this step, we obtained the number of medical students on Chinese government scholarship from 2007-2016.

Step 4: Generating estimate of MOE health aid volume

We multiple the number of medical students on Chinese government scholarship with the average cost for the specific year we obtained in step 2 and obtained the disbursement of MOE health aid volume from 2007-2016.

eTable 5 reports the estimate generation process discussed above.

eTable 5 Estimate generation for development assistance for health from the Ministry of Education

Year	All number of student receiving scholarship	Number of medical student receiving scholarship	Scholarship amount extracted from Department Account	Average cost per student	Average cost per medical student	Proportion of medical sciences students	Estimated number of medical science students	Estimated DAH through MOE
2007	10151	1131.101	302.5498	0.029805	0.0327854	0.111428	1131.101	37.08362
2008	13516	1409	498.2511	0.036864	0.0405502	0.104247	1409	57.1352
2009	18245	1841	656.044	0.035957	0.0395532	0.100904	1841	72.81747
2010	22390	2494.863	801.47	0.035796	0.0393755	0.111428	2494.863	98.23642
2011	25687	3317	919.8675	0.035811	0.0393917	0.129132	3317	130.6622
2012	28768	3205.548	1550.531	0.053898	0.0592876	0.111428	3205.548	190.0491
2013	33322	3712.989	1687.345	0.050638	0.0557013	0.111428	3712.989	206.8184
2014	36943	4116.468	1950.794	0.052806	0.0580861	0.111428	4116.468	239.1095
2015	40600	4523.959	2194.598	0.054054	0.0594596	0.111428	4523.958	268.9926
2016	49022	5462.401	2291.237	0.046739	0.0514129	0.111428	5462.401	280.8376
2017	58600	6529.654	2877.946	0.049112	0.0540229	0.111428	6529.654	352.7507

Note: Aid volume is expressed in 10,000 CNY.

eFigure 4 Trends in observed and estimates for development assistance for health from the Ministry of Education

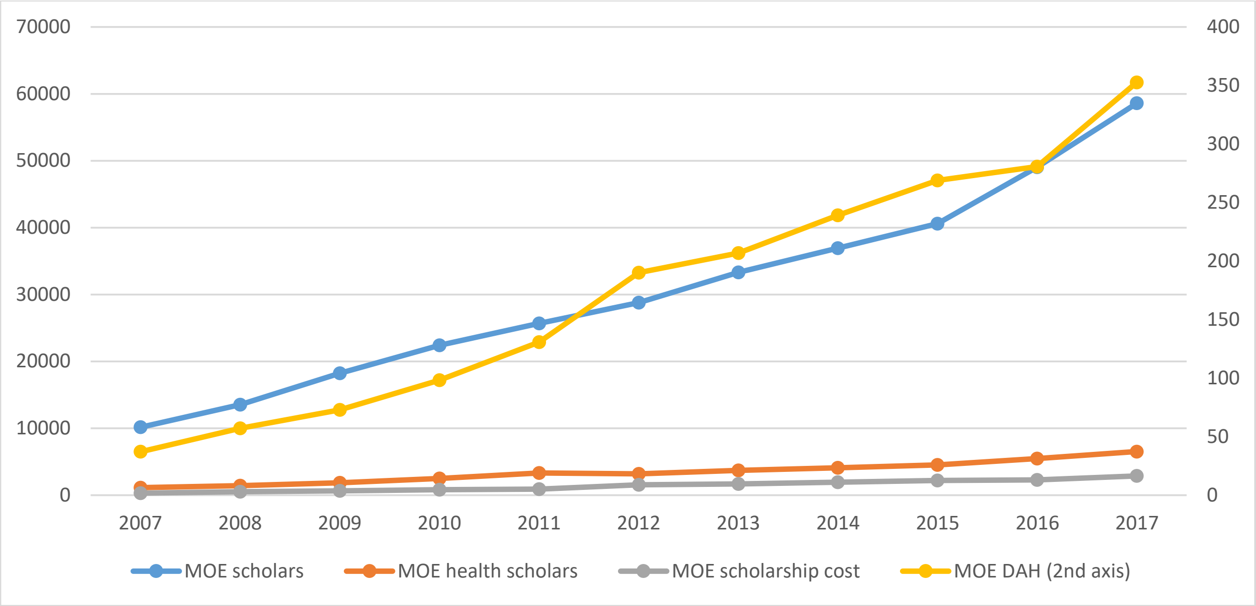

Note: Aid volume is expressed in 10,000 CNY.

Tracking development assistance for health from the Export-Import Bank (EXIM)

The Export-Import Bank of China Bank is the only policy bank in China that provides concessional loans (13).

Step 1: Extracting all EXIM concessional loan volume

We extracted the concessional loan disbursements from 2007 through 2008 and from 2010 through 2014 from the Almanac of China's Finance and Banking (43). The disbursement amount is reported in million USD, and so we converted the volume into CNY based on respective year's currency exchange rate extracted from the OECD exchange rate database. At this step, we are missing the following years of data: 2009, 2015, 2016, 2017.

Step 2: Generating estimate of health proportion of concessional loans disbursement

We extracted all the EXIM Bank's loan projects from 2000 to 2014 reported in the AidData database (44). We excluded all the preferential export buyer's credit projects, since these projects are export credit with certain preferential forms to promote export of capital goods and services, and are considered "other official flow" instead of official development assistance.

We also excluded those projects marked as "FALSE" in the database, a variable in the AidData database where the project either "is umbrella project", or "have not moved through the project cycle to at least the committed, implementation and completed stages". We manually screened all the projects to identify the health-related projects. We calculated the total volume of health-related projects and total volume of all remaining projects by year using the amount provided in the AidData database (within the dataset column "USD deflated 2014"). For multi-sector, a specific proportion of the projects was counted as health based on the project description (e.g., "another 6 million dollars will be used to equip a local hospital").

We then calculated the overall average share of disbursements related to health by averaging over all the annual shares available from 2000-2014. We obtained an estimate of 0.014773. We used this constant proportion for all years, i.e. 1.48% of all EXIM loans flow to health sector. See eTable 6 for a list of projects we included.

Step 3: Generating estimate of health concessional loan disbursement

We multiply the available concessional loan disbursement data extracted in Step 1 with 1.4773% to get a time series of health concessional loan disbursement from the EXIM Bank.

Step 4: Imputing missing years of data

We first filled the missing data points for 2009 using linear interpolation. This strategy was used here because they were missing data points between years for which we had data so we could interpolate by taking the average between those data points. We then imputed the remaining missing years of data – 2015-2017 - based on the growth rate. We used a difference in log health disbursements and year regression model to predict the growth rate for the missing years. eBox 3 above provides details on how this was estimated.

eBox 2 Regression output from growth rate estimation for Export-Import Bank of China

Source	SS	df	MS			
Model	.002696738	1	.002696738	Prob > F	=	0.8673
Residual	.435715714	5	.087143143	R-squared	=	0.0062
				Adj R-squared	=	-0.1926
Total	.438412452	6	.073068742	Root MSE	=	.2952

Number of obs = 7; F(1, 5) = 0.03

dlog_HEALT~H	Coef.	Std. Err.	t	P>|t|	[95% Conf.	Interval]
YEAR	-.0098139	.0557876	-0.18	0.867	-.1532204	.1335927
_cons	19.86042	112.1889	0.18	0.866	-268.5303	308.2512

We predicted growth rates for missing years and transformed the growth rate by adding 1 to ease the conversion of the rates to health estimates. We obtained DAH estimates for years with missing data in the future by multiplying the most recent year of data available by the transformed growth rate. For example, for 2015 the transformed growth rate was 0.0854671 and the value of the previous year (2014) with data was 199.4798. Our estimate for 2015 was 199.4798*0.0854671 = 216.5288 as shown in eTable 7 below.

eTable 7 reports the raw data and the results from the imputation methods discussed above.

eTable 6 List of projects from AidData Global Chinese Official Finance Dataset database included as health related projects for EXIM Bank proportion calculation

Project id	Year	Funding agency	Recipient condensed	Title	Description	Amount in USD (2014 deflated)
1863	2004	Export-Import Bank of China, Government Agency	Morocco	China Exim Bank loans to build six clinics in Morocco	In May 2004, China and Morocco signed the construction agreements on the 6 clinics. The 6 clinics are 1) Berechid, Settat 2) Benslimane, Benslimane, 3) Guercif, Taza, 4) Khmiss Zmamra, El Jadida, 5) Sidi Youssef Ben Ali, Marrackech, and 6) Ouled Taima, Taroudant. Each clinic has 45 beds and 1 house for the doctors, with the total construction area of 20,238 square meters. The project costs CNY 125,227,814.45 (all tax included), paid for by the CNY 150 million preferential loan that China EXIM bank has agreed to provide to Morocco in 1998. The construction completed on October 23, 2008.	31,179,770
34783	2005	Export-Import Bank of China, Government Agency	Angola	Construction and Equipping of 2 Kwanza-Norte Health Centers (linked to #42029)	On 29 October, 2005 a contract was signed between China and Angola for the construction and equipping of two health centers in Kwanza-Norte. The contractor was CAMCO and the value of the contract was 8,006,954.00 USD. The two parts of the contract began on 10 March and 25 March, 2006 and the two projected handover dates were 25 September (for Dondo city) and 10 October, 2007 (for Ndalatando city). The initial payment for this project was 800,695.40 USD and the amount financed by China as of December, 2007 was 7,206,258.60 USD. The number of Angola workers for this project was 62 and the number of Chinese workers was 47. This project received funding in September 2007 (See project #34831), and both hospitals were inaugurated by early 2010. This project is part of a larger credit line provided by China to Angola. It is part of Phase 1, which is the first 1 billion USD tranche of the 2 billion USD loan to Angola committed in 2004 (see project ID#42029). Please refer to the appendix in "Uncovering African Agency" by Lucy Corkin for further information.<br><br>STAFF_NOTE: In her Appendix, Corkin lists two health centers built in Kwanza-Norte and Kwanza-Sul. However, the documents from Angola's Ministry of Finance indicate that the two health centers were both located in Kwanza-Norte. This record reflects the Ministry of Finance's information.	14,154,007
34796	2005	Export-Import Bank of China, Government Agency	Angola	Rehabilitation and Equipping of Malange Central Hospital (linked to project ID #42029)	On 9 May, 2005 a contract was signed between China and Angola for the rehabilitation and equipping of a hospital in Malange (Malanje), reportedly located in Calculama city. The contractor was SinoHydro and the value of the contract was 29,185,499.00 USD. The contract began on 9 June, 2006 and the projected handover date for the project was 9 December, 2007. The initial payment for this project was 2,918,549.90 USD and the amount financed by China as of December 2007 was 26,266,949.10 USD. Other sources indicate that the project commenced March 9, 2006 and was completed August 3, 2008. This hospital should not be confused with the Malanje Municipal Health Center (See #34832). The seven-story building will cover an area of 54.4 thousand square meters,with areas for visits, hospitalizations, surgeries, mortuary, maternity, administrative services, dining and laundry. The project also provides for an oxygen plant, incinerator, area for generators, new water networks, sanitation, electricity and mechanics. Among the specialties are ophthalmology and dentistry. The hospital will be equipped with X-ray rooms, clinical laboratories, ultrasound and electrocardiogram. There will be 175 beds available for patients. This project is part of a larger credit line provided by China to Angola. It is part of Phase 1, which is the 1 billion USD tranche of the 2 billion USD loan to Angola committed in 2004 (see project ID#42029). Please refer to the appendix in 'Uncovering African Agency' by Lucy Corkin for further information.	51,591,628
34798	2005	Export-Import Bank of China, Government Agency	Angola	Rehabilitation and Equipping of Benguela Central Hospital (Linked to project ID #42029)	On 9 May, 2005 a contract was signed between China and Angola for the rehabilitation and equipping of Benguela Regional Hospital. The contractor was SinoHydro and the value of the contract was 40,521,731.00 USD. The contract began on 3 June, 2006 and the projected handover date for the project was 3 July, 2007; construction completed August 13, 2008. The initial payment for this project was 40,521,731 USD and the amount financed by China as of December 2007 was 36,469,557.90 USD. The hospital has 37 room and almost 400 beds. The parking lot contains 120 spaces.	71,630,848

					STAFF_NOTE: This project is part of a larger credit line provided by China to Angola. It is part of Phase 1, which is the 1 billion USD tranche of the 2 billion USD loan to Angola committed in 2004 (see project ID #42029).	
34836	2005	Export-Import Bank of China, Government Agency	Angola	Construction of Health Centers in Benguela and Huila (Linked to #42029)	May 9, 2005, the Chinese corporation Sinohydro signed a contract with Angola to construct and equip multiple municipal hospitals and health centers. The contract value was 43,805,500.00 USD. The hospitals were handed over Dec 15, 2008; the health centers were handed over Sep 5, 2008. The initial payment on the project was USD 4,380,550.00, and 89 Angolan workers and 73 Chinese workers were involved with this project. This contract refers to the construction of three municipal hospitals in Huambo, Huila, and Benguela, as well as two health centers in Benguela and two health centers in Huila. However, competing information indicates that all three municipal hospitals at Benguela, Huila, and Huambo were implemented under separate contracts with Sinohydro (#34798; #34843; #34844). As such the costs of construction for these three hospitals are presumably not covered by the transaction reported here. This transaction only pertains to the payment made for the two health centers in Benguela, and two health centers in Huila. This project is financed by the second tranche of the 2 billion USD credit facility to Angola in 2004 (#42029). The first billion was disbursed in 2004 and the second billion was disbursed in 2007. Staff_note:Please refer to the appendix in 'Uncovering African Agency: Angola_„Žs Management of China_„Žs Credit Lines' by Lucy Corkin for further information.	8,603,957
34842	2005	Export-Import Bank of China, Government Agency	Angola	Construction of Namibe Regional Hospital (Linked to #42029)	In October 2005 a $9.26 million contract was signed between SinoHydro and Angola for the construction and equipping of a regional hospital in Namibe. The funds were provided by Phase I, which is the $1 billion tranche of a $2 billion loan China's Exim Bank provided to Angola in 2004, which has a maturity period of 17 years, a 5 year grace period and an interest rate of 3.297 percent (#42029). Construction began in November 2006, and was completed in July 2007, but was not handed over to Angola until December 2008. There were a total of 40 Angolan and 35 Chinese workers.	18,195,407
34843	2005	Export-Import Bank of China, Government Agency	Angola	Construction and Equipping of Huila Hospital and Health Centers (linked to #42029, #34030)	On 24 October, 2005 a contract was signed between China and Angola for construction and the equipping of a regional hospital in Lubango city, HuÕla province. The contractor was SinoHydro and the value of the contract was 48,060,432.43 USD. The contract began on 10 July, 2006 and the projected handover date for the project was 10 January, 2008. The initial payment for this project was 4,806,043.24 USD and the amount financed by China as of December 2007 was 43,254,389.19 USD. The number of Angolan workers for this project was 127 and the number of Chinese workers was 68. Accordingly this building's construction ended June 30, 2008, and housing for doctors was underway afterwards.<br><br>This project is part of a larger credit line provided by China to Angola. It is part of Phase 2, which is the second 1 billion USD tranche of the 2 billion USD loan to Angola committed in 2004 (see project ID#991). The project is also linked to #34030 because of the 500M transaction that was later added to the original 2 billion Chinese loan commitment to Angola.	84,957,121
34844	2005	Export-Import Bank of China, Government Agency	Angola	Rehabilitation and Equipping of Huambo Central Hospital in Angola (linked to #42029)	On 24 October, 2005, a contract was signed between China and Angola for rehabilitation and equipping of Huambo Central Hospital and for the provision of 86 ambulances. The contractor was SinoHydro and the value of the contract was 36,520,308.99 USD. The sections of the contract began on 1 August, 2006 and 21 April, 2007 and the projected handover date for the project was 1 Feburary, 2008. The initial payment for this project was 3,652,030.90 USD and the amount financed by China as of December 2007 was 32,868,278.09 USD. There were 57 Angolan workers and 30 Chinese workers for this project. The hospital had a ribbon cutting ceremony on November 11, 2009. This project is part of a larger credit line provided by China to Angola.<br><br>STAFF_NOTE: This is part of Phase 1 of Project ID #42029 which is the 1 billion USD tranche of the 2 billion USD loan to Angola committed in 2004.	71,730,532
34846	2005	Export-Import Bank of China, Government Agency	Angola	Construction and Equipping of Two Municipal Hospitals in Kwanza-Norte and Kwanza-Sul (Linked to project IDs #42029, #34030)	On 29 October, 2005 a contract was signed between China and Angola for the construction and equipping of two municipal hospitals in Cazengo, Kwanza-Norte Province and Sumbe, Kwanza-Sul Province. The contractor was with CAMCO and the value of the contract was 18,527,728.00 USD. The sections of the contract began on 17 November and 1 November, 2006 and the projected handover dates for the project were 17 February and 1 February, 2008. The initial payment for this project was 1,852,772.80 USD and the amount financed by China as of December 2007 was 16,674,955.20 USD. There were 25 Angolan workers and 20 Chinese workers for this project. This project is distinct from the the two health centers built in Kwanza-Norte (#34783). This project is part of a larger credit line provided by China to Angola. It is part of Phase 1, which is the 1 billion USD tranche of the 2 billion USD loan to Angola committed in 2004 (see project ID#42029). Please refer to the appendix in 'Uncovering African Agency' by Lucy Corkin for further information.The project received additional funding as part of a 500 million USD extension (See #34831) to the credit line given to Angola by China EXIM in September 2007 (#34030).	32,751,732

34845	2005	Export-Import Bank of China, Government Agency	Angola	Construction and Equipping of Huambo Health Center (linked to #42029 & #34030)	On 24 October, 2005 a contract was signed between China and Angola for the construction and equipping of a health center in Huambo province, located in Bairro de Sao Pedro, Kasseque II municipality. The contractor was SinoHydro and the value of the contract was 4,003,477.00 USD. The contract began on 1 August, 2006 and the projected handover date for the project was 1 August, 2007. The initial payment for this project was 400,347.70 USD and the amount financed by China as of December 2007 was 3,603,129.30 USD. There were 30 Angolan workers and 25 Chinese workers for this project. This project also received additional funding in September 2007 through a 500 million credit line extension from EXIM bank to Angola (See project ID #34030 and #34841).This is not to be confused with the Huambo Central Hospital (#34844).<br><br>The project is also linked to Project ID #42029 because it was financed by the USD2B loan in 2004. However, it is unclear if it is part of Phase 1 or Phase 2.	7,077,004
34830	2007	Export-Import Bank of China, Government Agency	Angola	Complementary Action (#34030): Four Regional Hospitals	In September 2007, China EXIM Bank provided Angola with a loan to finance a project involving four regional hospitals in Malange, Benguela, HuÍ_la, and Huambo with contractor SinoHydro (See projects #34796, #34798, #34843 and #34845 respectively). The loan was worth 75,917,724.36 USD. As of June 30, 2008, the project's required documents were sent to China EXIM Bank and the project was awaiting disbursement. This project is part of the USD 500 million loan provided to Angola by China on September 28, 2007 (Project ID #34030). Please refer to the appendix in 'Uncovering African Agency' by Lucy Corkin for further information.	123,558,518
34832	2007	Export-Import Bank of China, Government Agency	Angola	Conversion of Health Center into Municipal Hospital in Caculama (Linked to #34030 & #34841)	In September 2007, China EXIM Bank provided Angola with a loan to provide additional funding for the municipal hospital in Caculama city, Malanje (Malange) Province. The loan was worth 10,875,000 USD and the project was made with contractor SinoHydro. As of June 30, 2008, the project's required documents had been sent to China EXIM Bank and was awaiting disbursement of funds. The hospital was also inaugurated in 2008 and was operational in 2014, although it suffers from staff, energy, and water shortages occasionally. This hospital should not be confused with the General Hospital of Malanje (Malanje National Hospital), which is also located in located in Caculama and also constructed by SinoHydro (see project #34796). Rather, the Caculama Municipal Hospital serves to relieve traffic at the general hospital. This project is part of the USD 500 million credit line provided to Angola by China in 2007 (Project ID #34030). Please refer to the appendix in 'Uncovering African Agency' by Lucy Corkin for further information.	17,699,409
41898	2008	Export-Import Bank of China, Government Agency	Samoa	Chinese EXIM Bank gives $41m loan to Samoa for the construction of a National Medical Centre and Ministry of Health Headquarters	In 2008 construction began on the Samoan National Medical Centre and Ministry of Health Headquarters. The venture is being funded by the Chinese EXIM bank with a concessional loan of $41m with an interest rate of 2%, a 5 year grace period and a 20 year maturity rate. The Chinese handed over the buildings to the Samoan government on July 23, 2011	56,535,437
2324	2010	Export-Import Bank of China, Government Agency	Zambia	China gives Zambia 361 million CNY Concessional Loan for mobile hospitals	In 2010, the Chinese government (through the EXIM bank) gave Zambia a 361 million Yuan concessional loan for the construction of mobile hospitals in the country. In April of 2011, the project was completed. The hospitals will play a large role in reducing maternal mortality rates and will bring health services closer to those who may not otherwise have access to medical facilities. The project was criticized since the contract was awarded to the implementing agency, China National Aero-Technology Import and Export Corporation (CATIC), without an open bidding process. STAFF_NOTE: According to a 2011 report on Chinese Foreign Aid from the State Council and a presentation given by China Export-Import Bank, EXIM bank offer concessional loans at an interest rate between 2 and 3% with a maturity of 15-20 years and a 5-7 year grace period. The borrower is expected to make two loan repayments per year. Imputing these criteria into the OECD's grant element calculator yields a grant element of at least 40%. As a result this projects has been coded as ODA-like.	67,062,181
35960	2010	Export-Import Bank of China, Government Agency	Bolivia	China Loans $67.8 Million USD to Bolivia for Regional Development	In 2010, the Chinese government provided Bolivia with a $67 million loan to build infrastructure in the mineral-rich department of Oruro. A total of 45 million dollars will be used to construct the port and build infrastructure, including transportation services as well as facilities for gathering, distributing, exporting and importing goods, Oruro's Mayor Alberto Aguilar said. Another 6 million dollars will be used to equip a local hospital, and the rest of the credit will be used to drill water wells, repair roads and build other projects.	84,265,772
1290	2011	Export-Import Bank of China, Government Agency	Kenya	China provides a $9.5 Billion KES concessional loan for the construction of Kenyatta University Teaching, Research and Referral Hospital (Linked to Project ID #34174)	In June 2011, China's Exim Bank provided a 9.5 billion KES concessional loan to Kenya for the construction of Kenyatta University Teaching, Research and Referral Hospital in Nairobi. Construction for the 600-bed hospital will involve expansion and equipping of anatomy and basic science laboratories and establishment of pre-clinical and clinical teaching departments at the university. In addition, the project will establish specialized research and training departments of cancer, neurosciences and alternative medicine that are not being addressed by any institution of higher learning in Kenya. Construction began in 2011, and was completed in June 2014.	123,108,663

22062	2011	Export-Import Bank of China, Government Agency	Zimbabwe	China provides $99.5 million loan to Zimbabwe for medicine	In 2011, China's Exim Bank provided a $99.5 million loan to Zimbabwe for the procurement of medicine. The agreement was signed as part of financing package totaling US$585 million. Chinese Vice Prime Minister Wang Qishan, Vice Minister of Commerce Fu Zi-ying, President of the Export-Import Bank of China Li Riougut, and Zimbabwe's Minister of Finance Tendai Biti attended the signing ceremony.	110,443,739
39716	2013	Export-Import Bank of China, Government Agency	Trinidad & Tobago	China completed the construction of Trinidad and Tobago Children's Hospital with concessional loan of $950 million [linked to #39884]	On Dec. 28, 2012, China offered Trinidad and Tobago $150 million concessional loan for developments works at the proposed Children's Hospital in Couva. The 230-bed hospital would be located on a site near Preysal, Central Trinidad. The project, implemented by Shanghai Construction Group, started on June 1, 2013 and was completed on Aug 14, 2015.	152,513,005
41591	2014	Export-Import Bank of China, Government Agency	Zimbabwe	China Exim Bank loans Zimbabwe 89 million USD to retool healthcare equipment	On 17 July 2014, China Exim Bank loaned $89 million to Zimbabwe for the purchase of healthcare equipment, including CT scan machines, Magnetic Resonance Imaging machines, dialysis machines and their consumables, ultra sound machines, digital x-ray machines, oxygen generating machines and baby incubators.Provincial hospitals will for the first time offer services that they never used to offer such as dialysis and cancer diagnosis, lowering the demand on more populated facilities elsewhere. The loan will attract a two percent interest rate and 0,5 percent management and commissioning rates payable over 20 years with a grace period of five years.	89,000,000
47032	2014	Export-Import Bank of China, Government Agency	Mongolia	China Exim Bank Loans Mongolia $14.9 Million USD for Medical Information Systems	In August 2014, a loan agreement on an _„ñE-Health_„• project was signed between the Ministry of Economic Development of Mongolia and the Export-Import Bank of China. China will provide the $14.9 million USD loan to create information technology systems for medical organizations and hospitals. In 2016 the opening ceremony for this project took place.	14,900,000

eTable 7 Estimate generation for development assistance for health from the Export-Import Bank

Year	Concessional loan in million CNY	Health Concessional loan in million CNY	Growth rate	Estimated DAH through EXIM after imputation
2007	5639.83	83.31721	1.758712	83.31721
2008	4342	64.14436	.738481	64.14436
2009		112.4447#	1.561325	112.4447
2010	10881	160.745	1.357358	160.745
2011	12073	178.3544	1.103953	178.3544
2012	11164	164.9258	.9217229	164.9258
2013	11994	177.1874	1.071712	177.1874
2014	13503	199.4798	1.118505	199.4798
2015			1.085467*	216.5288#
2016			1.075653*	232.9099#
2017			1.056026*	248.2445#

Note: The growth rate marked “*” are predicted based on linear regression, the growth rate marked “#” are predicted based on interpolation.

eFigure 5 Trends in observed and estimates of development assistance for health from the Export-Import bank

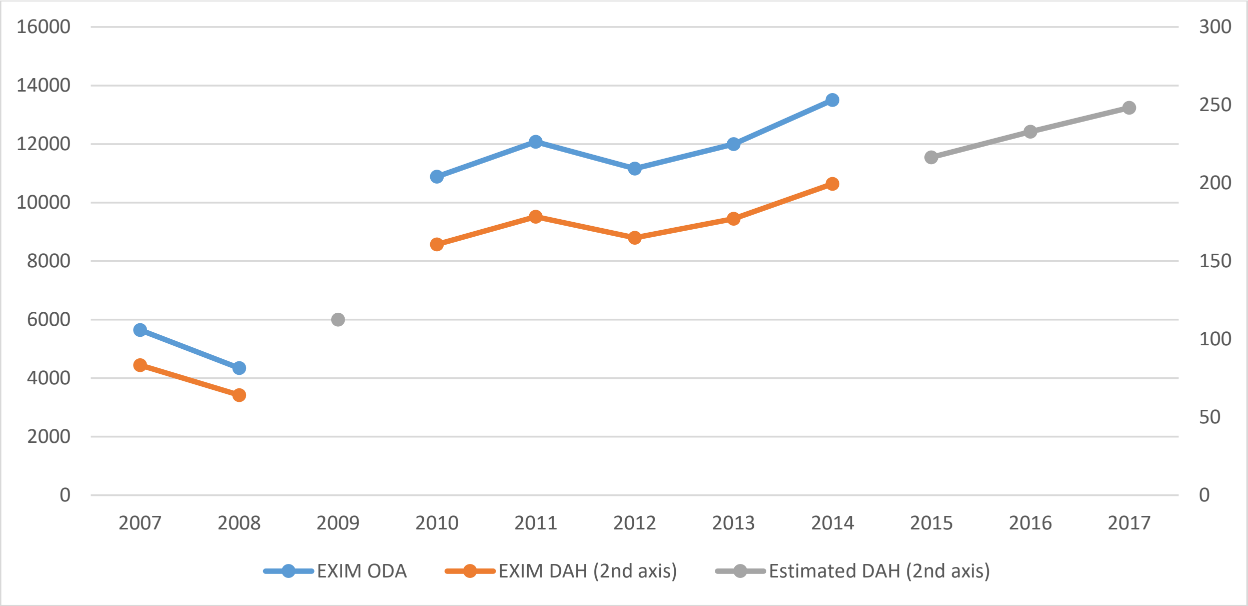

Note: Aid volume is expressed in 10,000 CNY.

Calculating the administrative cost contribution for China's bilateral agencies

We also calculated the administrative cost contribution for China for all the bilateral channels. We defined administrative cost contribution as the costs associated with administering grants and loans. Given that the Ministry of Commerce's Department of Foreign Aid is the largest agency managing development aid and could best reflect the administrative condition of China's development assistance for health, we used the administrative cost ratio of the Ministry of Commerce's as a proxy for all the bilateral agencies, and generated the administrative cost ratios through calculating the basic expenditure for foreign affairs over project expenditure for foreign affairs in the annual department account (15). We used the year-specific ratio for 2007-2017. See eTable 8 for the estimate for the administrative cost ratio estimate.

eTable 8 Estimate generation for the administrative cost contribution for China's bilateral agencies

Year	Basic expenditure for foreign affairs for MOFCOM	Project expenditure for foreign affairs for MOFCOM	Administrative cost ratio
2007	86697.69	1110576	0.078066
2008	84035.37	1190224	0.070605
2009	86943.07	1335686	0.065092
2010	86064.64	1495957	0.057531
2011	127668	1805137	0.070725
2012	143812.4	2115060	0.067994
2013	100114.6	1971741	0.050775
2014	103838	1908830	0.054399
2015	110979.7	1707390	0.065
2016	116167.2	1829605	0.063493
2017	141017.2	2085405	0.067621

Note: Aid volume is expressed in 10,000 CNY.

Part 3. Estimating DAH from China Through Multilateral Organizations and Public-Private Partnerships

We used data from IHME's forthcoming Financing Global Health 2018 Development Assistance for Health database to estimate Chinese disbursements to multilateral aid agencies (45). We identified the following channels as having received contributions from China: the United Nations Population Fund (UNFPA), the Joint United Nations Programme on HIV/AIDS (UNAIDS), the United Nations Children's Fund (UNICEF), WHO, the World Bank, the Asian Development Bank, the African Development Bank, the Inter-American Development Bank, the Global Fund, and Gavi. This particular study did not include China's contribution to NGOs.

Besides the World Bank, the methodology used to estimate DAH contributions from China are the same as reported in the annex of the Financing Global Health report. Additional details on methodology for those multilateral agencies can thus be found in IHME's Financing Global Health 2018 Supplementary Methods Annex at http://www.healthdata.org/sites/default/files/files/policy_report/FGH/2019/FGH2018_Methods-Annex.pdf

United Nations Agencies: Page 84
The Global Fund and Gavi: Page 81
The World Bank: Page 75
Regional Development Banks: Page 77
Calculating technical assistance and program support for loan- and grant-making channels: Page 93

Below we provide additional detail on how we generated estimates of DAH contributions from China disbursed through the World Bank.

Tracking development assistance for health from the World Bank

The World Bank project-level health disbursement data for 2000 through 2017 were obtained through correspondence with Kathleen E. Krackenberger. The World Bank recently underwent a recoding process for their disbursements. This recoding affected health disbursements; however, the recoding was not completed for projects with disbursements prior to 2001. To create a comparable dataset, adjustments had to be made. Regression analysis to predict health disbursements was explored; however, in the end, the average percent change between project-level health disbursements before and after recoding was used to adjust health disbursements prior to 2001. It was observed that on average, between 2001 and 2005 (inclusive) the recoding process decreased health disbursements by 0.22%. This number was used to adjust all project-level health disbursements prior to 2001.

Health disbursements included all health projects as well as other sector projects with a health sector code. In addition, data were collected from the World Bank online loans database in order to fill in descriptive information for loans from the two arms of the World Bank: the International Development Association (IDA) and the International Bank for Reconstruction and Development (IBRD). Along with keyword searches, health theme codes were used to allocate disbursements by health focus area. The online database contains up to five sector codes and five theme codes that can be assigned to each project. Sector codes represent economic, political, and social subdivisions, while theme codes represent the goals or objectives of World Bank activities. The codes are summarized in eTable 9. Emergency recovery loans were excluded since they do not fit the definition of DAH.

eTable 9 World Bank's health sector and theme codes

**Health sector codes**<br>Sector codes represent economic, political, or social subdivisions within society. World Bank projects are classified by up to five sectors.	**Health theme codes**<br>Theme codes represent the goals or objectives of World Bank activities.
Historical (prior to 2001):<br>(1) Basic health<br>(2) Other population health and nutrition<br>(3) Targeted health<br>(4) Primary health, including reproductive health, child health, and health promotion<br><br>Current (as of 2001):<br>(1) Health<br>(2) Compulsory health finance<br>(3) Public administration – health<br>(4) Noncompulsory health finance	Current:<br>(1) HIV/AIDS<br>(2) Malaria<br>(3) Tuberculosis<br>(4) Other communicable diseases<br>(5) Population and reproductive health<br>(6) Child health<br>(7) Nutrition and food security<br>(8) Injuries and non-communicable diseases<br>(9) Health system performance<br>(10) Social analysis and monitoring

Data on yearly government contributions were obtained from the Development Assistance Committee (DAC) statistics in order to disaggregate IDA flows by source. Because China does not report to DAC, we generated contribution estimates using replenishment data. We split the 3-year replenishment amount over the three years of the 16th-19th replenishment, to obtain China's contributions to the World Bank from 2008-2017 (47). We validated our use of replenishment data by extracting China's and other countries' contributions to the World Bank International Development Association (IDA) 16th, 17th and 18th replenishment and comparing the number with the contribution we extracted from the OECD Creditor's Reporting System (CRS). The trend of contribution from major donors was similar, we therefore used China's replenishment contribution as a proxy of the contribution to World Bank IDA.

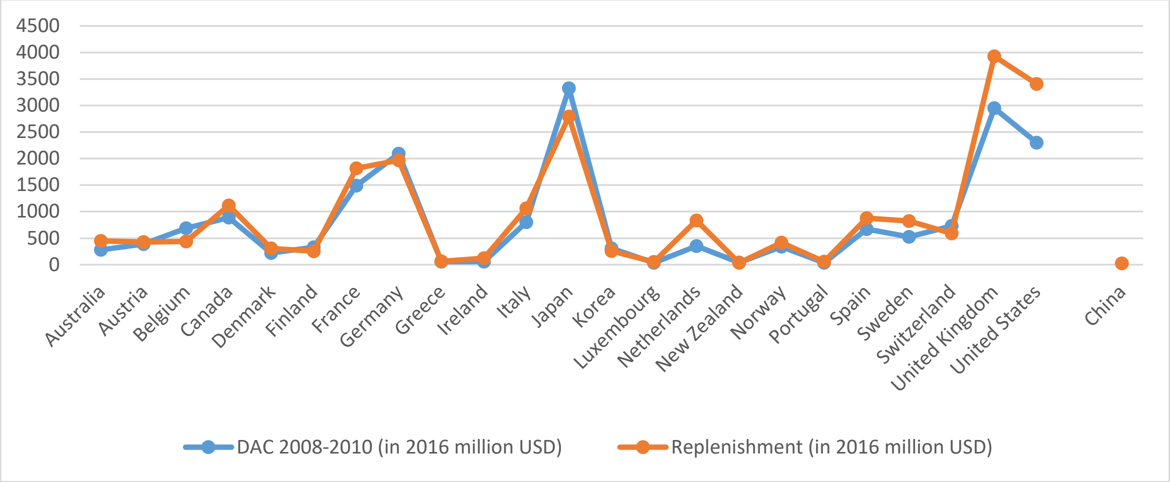

Panel a. Comparison of World Bank International Development Association 16th Replenishment

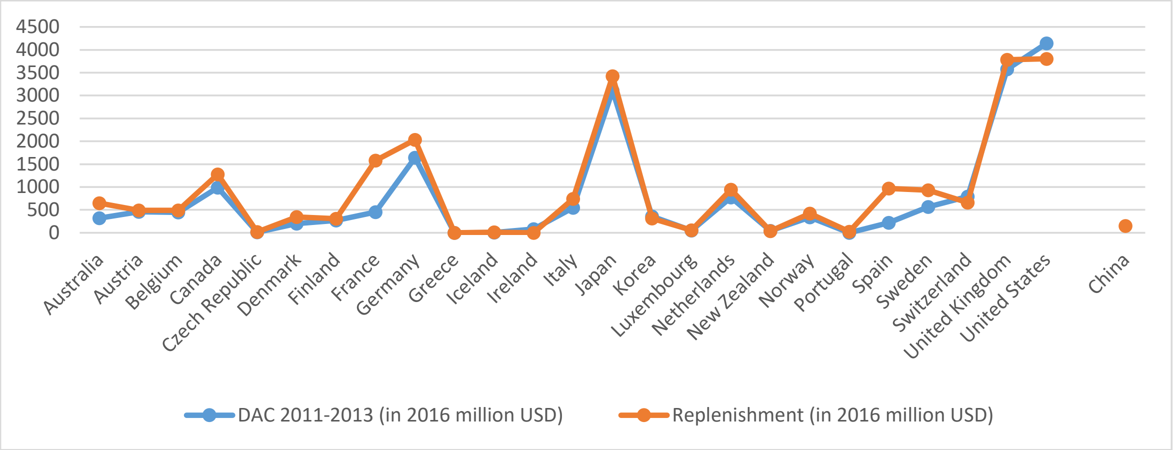

Panel b. Comparison of World Bank International Development Association 17th Replenishment

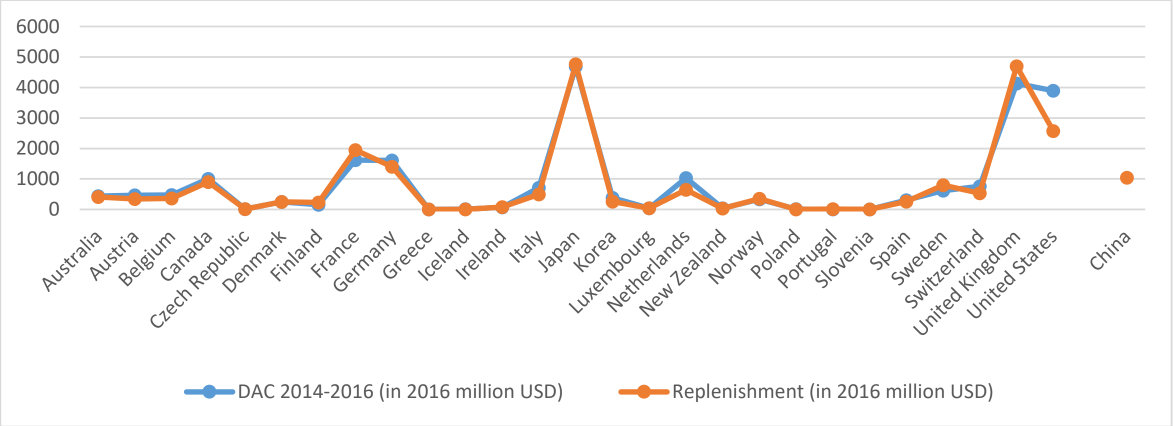

Panel c. Comparison of World Bank International Development Association 18th Replenishment

eFigure 6 Comparison of World Bank IDA Replenishment and DAC

The three figures show the comparison of country commitment to World Bank IDA replenishment rounds, and yearly government contributions reported to DAC statistics. The orange line shows the country commitment to each IDA

replenishment round. The blue line shows the sum of yearly reported contribution during the three-year replenishment round. Panel a is contributions and commitments from replenishment round 16; panel b is contributions and commitments from replenishment round 17; panel c is contributions and commitments from replenishment round 18.

***eTable* 10 World Estimate generation for development assistance for health through World Bank IDA**

Year	Channel	Donor Name	Donor annual contribution	Total donor contribution	Donor share	Channel disbursement	Donor-specific disbursement	Donor-specific administrative cost disbursement	Total
2008	WB_IDA	China	9.20	11806.25	0.08%	547.97	0.43	0.06	0.48
2009	WB_IDA	China	9.20	9260.75	0.10%	804.07	0.80	0.11	0.91
2010	WB_IDA	China	9.20	8772.08	0.10%	735.66	0.77	0.10	0.87
2011	WB_IDA	China	50.02	11908.27	0.42%	875.13	3.68	0.57	4.24
2012	WB_IDA	China	50.02	10225.89	0.49%	765.71	3.75	0.50	4.24
2013	WB_IDA	China	50.02	10820.12	0.46%	907.95	4.20	0.63	4.82
2014	WB_IDA	China	347.64	16984.08	2.05%	720.81	14.75	1.81	16.56
2015	WB_IDA	China	347.64	11387.66	3.05%	861.66	26.30	3.13	29.44
2016	WB_IDA	China	347.64	10949.69	3.17%	849.46	26.97	2.81	29.78
2017	WB_IDA	China	200.00	12137.46	1.65%	1349.84	22.24	3.14	25.38

Note: Aid volume is expressed in millions of current US dollar.

Part 4. Aggregating China's total DAH contribution and disaggregating the health focus area

For the four bilateral channels, we converted the volume in Chinese Renminbi (CNY) into US dollar (USD) based on year specific exchange rates extracted from the OECD exchange rate database (48). We further deflated all channel disbursements to constant 2017 USD using the International Monetary Fund deflator series (49). We finally aggregated China's total DAH contribution by adding up the previously estimated bilateral and multilateral contributions.

Estimating the health focus area of DAH from China

We also categorized DAH from China into health focus areas. Health focus areas of interest include those used in previous research, including HIV/AIDS, malaria, tuberculosis, reproductive and maternal health, newborn and child health, other infectious diseases, non-communicable diseases, health system strengthening, and other. We disaggregated the multilateral channels using the same methodology as in Chang et al.(50). For a more detailed step-by-step explanation, please refer to IHME's Financing Global Health 2018 Supplementary Methods Annex at http://www.healthdata.org/sites/default/files/files/policy_report/FGH/2019/FGH2018_Methods-Annex.pdf

Health focus area: Page 49

Due to lack of project-level information for the bilateral channels, we were only able to allocate the health focus area based on available literature and other information included in the Department Final Accounts. We allocated the National Health Commission's DAH to health system strengthening and other based on the amount of "medical aid (which footnoted as medical team provision") and "other types of aid", listed in the Department Final Account (18). We allocated the Ministry of Commerce and Export-Import Bank's DAH to health system strengthening considering its projects as described in the Commerce Yearbook are health infrastructure, equipment and medical products provision (14); We allocated the Ministry of Education's DAH also to health system strengthening because the scholarship for medical students support human resource for health (16).

Part 5. Comparing DAH from China with other traditional donor countries and previous estimates

We compared DAH from China with other traditional donor countries that are members of the development assistance committee of the Organisation for Economic Co-operation and Development (OECD) to highlight the global context of DAH contributions. We extracted DAH contributions from 23 traditional donor countries from IHME's Financing Global Health 2018 Development Assistance for Health database (51). We also extracted the population size, gross domestic product (GDP), and total government spending data from IHME's Financing Global Health and Global Burden of Disease database and calculated per person contribution, and per government spending for comparison. We also calculated the percentage of DAH flows to health system strengthening using health focus area information. We summed health system strengthening and all disease-specific health system strengthening (HSS) (HIV/AIDS-HSS, malaria-HSS, tuberculosis-HSS, reproductive and maternal health-HSS, newborn and child health-HSS, etc.) spending to generate our numerator of health system strengthening.

Furthermore, in recognition that China is still a developing country, we used an ordinary least square (OLS) regression analysis to examine donor DAH contributions based on income. We run a simple OLS regression excluding DAH contributions from China and used the fitted values from this regression to estimate the average expected level of contributions for all donor countries controlling for their gross domestic product per person. These fitted values are compared with the observed share of contributions. Countries who have observed values above the fitted line contribute more DAH than expected relative to their level of economic development. Alternatively, countries with observed values below the fitted line contribute less DAH than would be expected relative to their income level. We used the log of DAH contributions as a share of country GDP as the dependent variable and the log of lagged GDP per person as the independent variable. We lagged GDP per person to reflect that the translation of income growth to development expenditure is usually not instantaneous. The regression output is provided below. Stata version 13 was used to conduct this analysis.

eBox 3 Regression output from ordinary least square regression analysis

```
.                  reg log_dah_gdp log_laggdppc

      Source |       SS           df       MS      Number of obs   =        69
-------------+----------------------------------   F(1, 67)        =     70.98
       Model |  23.6919064         1  23.6919064   Prob > F        =    0.0000
    Residual |  22.3634725        67  .333783172   R-squared       =    0.5144
-------------+----------------------------------   Adj R-squared   =    0.5072
       Total |  46.0553789        68  .677284984   Root MSE        =    .57774

------------------------------------------------------------------------------
 log_dah_gdp |      Coef.   Std. Err.      t    P>|t|     [95% Conf. Interval]
-------------+----------------------------------------------------------------
log_laggdppc |   1.427963    .169492     8.42   0.000     1.089655     1.76627
       _cons |  -23.71168   1.858037   -12.76   0.000    -27.42034   -20.00302
------------------------------------------------------------------------------
```

Part 6. Comparing DAH with total official development assistance

eTable 11 Comparing official development assistance and development assistance for health from major donors in 2010-2016

		2010			2011			2012			2013			2014			2015			2016			2017		
Donor Name	ODA source	ODA	DAH	DAH/ ODA	ODA	DAH	DAH/ ODA	ODA	DAH	DAH/ ODA	ODA	DAH	DAH/ ODA	ODA	DAH	DAH/ ODA	ODA	DAH	DAH/ ODA	ODA	DAH	DAH/ ODA	ODA	DAH	DAH/ ODA
Australia	OECD	3778	641	17%	4851	804	17%	5138	900	18%	4504	811	18%	3727	804	22%	2897	442	15%	2388	504	21%	2468	376	15%
Austria	OECD	765	114	15%	607	138	23%	780	163	21%	722	103	14%	716	84	12%	867	67	8%	1123	89	8%	653	74	11%
Belgium	OECD	2385	359	15%	2171	361	17%	1484	329	22%	1531	336	22%	1554	370	24%	1386	281	20%	1467	295	20%	1517	285	19%
Canada	OECD	4489	1023	23%	4827	1104	23%	3693	1159	31%	3559	1388	39%	3758	1136	30%	3783	839	22%	4072	1097	27%	2760	1129	41%
Switzerland	OECD	2180	160	7%	3100	239	8%	2855	174	6%	4065	226	6%	3466	312	9%	2772	247	9%	2494	256	10%	3000	282	9%
China	OECD	2947	434	15%	3135	558	18%	3450	626	18%	3253	587	18%	3623	611	17%	3281	568	17%	3769	573	15%		652	
	AidData	28013	434	2%	53306	558	1%	43527	626	1%	39711	587	1%	39716	611	2%		568			573			652	
	JICA	4481	434	10%	5383	558	10%	5792	626	11%	6062	587	10%	5723	611	11%	6990	568	8%	6905	573	8%		652	
	Gov	2311	434	19%	2772	558	20%	2921	626	21%	2988	587	20%	3201	611	19%	3307	568	17%	2467	573	23%	2558	652	25%
Germany	OECD	12906	1250	10%	14086	1211	9%	13534	1129	8%	15288	1271	8%	19134	1401	7%	18608	1234	7%	25697	1552	6%	26670	1526	6%
Denmark	OECD	1994	409	20%	2388	376	16%	2130	310	15%	2204	282	13%	2205	281	13%	1962	240	12%	1575	202	13%	1576	230	15%
Spain	OECD	5389	717	13%	2988	437	15%	1260	191	15%	1029	205	20%	940	191	20%	727	127	18%	3058	199	7%	1135	225	20%
Finland	OECD	1240	195	16%	1330	179	13%	902	147	16%	940	190	20%	867	221	25%	607	132	22%	503	85	17%	658	88	13%
France	OECD	12697	1070	8%	10836	985	9%	12592	1085	9%	9714	1196	12%	9663	1252	13%	9139	754	8%	8721	1079	12%	10935	1086	10%
United Kingdom	OECD	9612	2418	25%	9923	3266	33%	9922	3119	31%	6800	3503	52%	7180	3658	51%	9561	4013	42%	7624	2919	38%	7468	3551	48%
Greece	OECD	243	21	8%	173	16	9%	119	12	10%	47	10	21%	49	18	36%	76	12	16%	166	12	7%	87	16	18%
Ireland	OECD	673	214	32%	680	172	25%	592	152	26%	592	161	27%	553	171	31%	451	133	29%	445	145	33%	504	150	30%
Italy	OECD	1083	251	23%	2255	262	12%	941	182	19%	999	226	23%	1633	270	17%	2111	281	13%	2656	353	13%	3508	368	10%
Japan	OECD	20129	1101	5%	18221	944	5%	19114	1258	7%	23427	815	3%	17422	932	5%	20594	930	5%	21920	1111	5%	20644	971	5%
Korea	OECD	2080	206	10%	1828	157	9%	1936	235	12%	2430	226	9%	2534	249	10%	2435	267	11%	2563	285	11%	2501	285	11%
Luxembourg	OECD	301	94	31%	316	86	27%	309	76	24%	328	94	29%	320	88	27%	274	70	26%	288	85	30%	314	78	25%
Netherlands	OECD	7125	828	12%	4532	806	18%	5386	644	12%	3602	781	22%	4222	696	16%	5412	693	13%	3412	790	23%	3538	643	18%
Norway	OECD	3853	680	18%	4210	741	18%	3893	672	17%	5010	801	16%	4519	879	19%	4018	727	18%	3605	643	18%	3314	661	20%
New Zealand	OECD	312	51	16%	371	47	13%	399	45	11%	381	48	13%	436	55	13%	377	34	9%	376	34	9%	379	32	9%
Portugal	OECD	496	37	7%	580	39	7%	444	30	7%	345	33	10%	297	35	12%	189	38	20%	165	28	17%	149	28	19%
Sweden	OECD	3375	646	19%	4114	654	16%	4027	672	17%	4253	735	17%	4670	680	15%	4552	596	13%	3407	709	21%	3668	673	18%
United States	OECD	33803	11999	35%	33195	13579	41%	27571	13802	50%	30289	13840	46%	30216	13058	43%	31607	12590	40%	32442	13422	41%	31787	14424	45%

Note: Aid volume is expressed in millions of 2018 USD

We compared DAH from China and other donor countries with their respective total official development assistance (ODA) data. We extracted the total ODA provided by DAC donors from the DAC5 table of OECD (52). While there is a lack of official ODA provided by China, we used four different sources to provided ODA estimates, including (1) estimates from OECD's annual development cooperation reports which covers year 2010-2016 (53); (2) estimates from AidData which covered 2010-2014 (44); (3) estimates from the Japan International Cooperation Agency Research Institute that covered 2010-2016 (3,4); (4) amount of foreign aid disbursement from the Finance Yearbook of China that covered 2010-2017 (37).

AidData provided the largest amount of ODA for China thus DAH as a percentage of ODA remained low from 1-2%. The other three data sources have more comparable ODA amounts for China, and DAH/ODA ranged from 10-19% in 2010, to 11-19% in 2014, and 8-15% in 2016. When compared with other DAC donors, the United States and the United Kingdom provided one-thirds or even half of their ODA to health projects, while there are also countries like Japan, and Switzerland that provided less than 10% of their ODA to health.

Part 7. Sensitivity analysis for China's development assistance for health

We conducted a sensitivity analysis for China's development assistance for health to examine the robustness of our results to the assumptions made in our analysis. For each of our bilateral channels, we conducted further analysis to estimate the lower bound of DAH provided and higher bound.

Scenario: Vary share of ODA through NHC by plus or minus 1 standard deviation and assume all NHC ODA is health related.

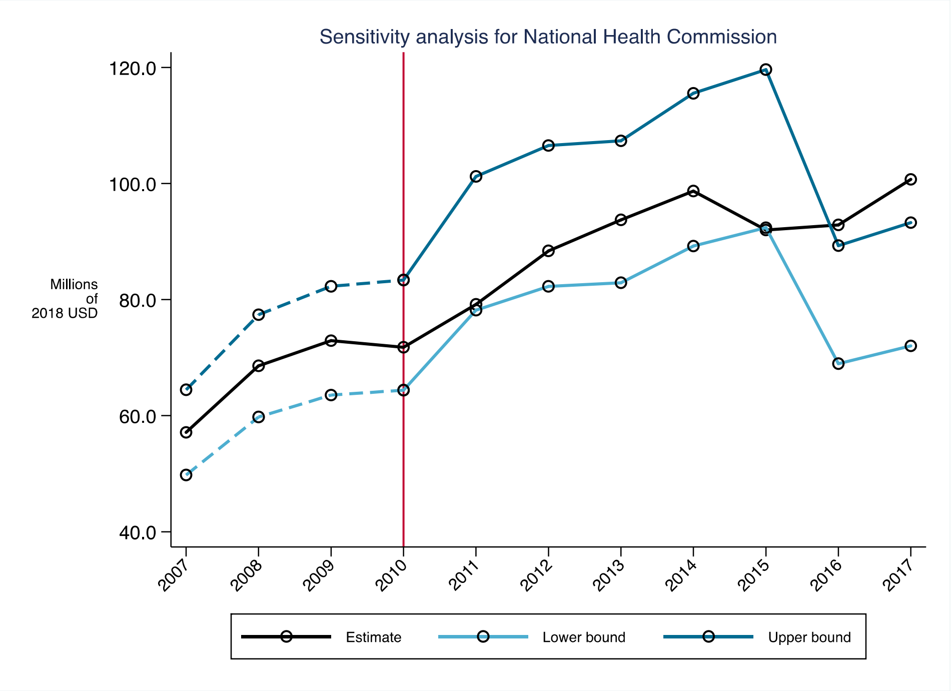

eFigure 7 Uncertainty level for China National Health Commission for the proportion of total government aid flows to National Health Commission

For National Health Commission, our estimate assumed that 3.0% of China's total development assistance flowed through National Health Commission for years before 2010, and the percentage was based on average for 2010-2017. We calculated the standard deviation of the percentage, and calculated the lower bound (2.6%) and upper bound (3.4%) through plus and minus one standard deviation of the proportion. As shown in eFigure 7, the dash lines from 2007-2010 show the modelled upper and lower bound of DAH provided through the National Health Commission, and the data points after 2010 are not modeled, the solid line of lower bound and upper bound are for reference. The range in estimates prior to 2010 is modest except for the last two years for which the bound is slightly widened.

Scenario: Vary share of MOFCOM ODA that is health related 10%-20%

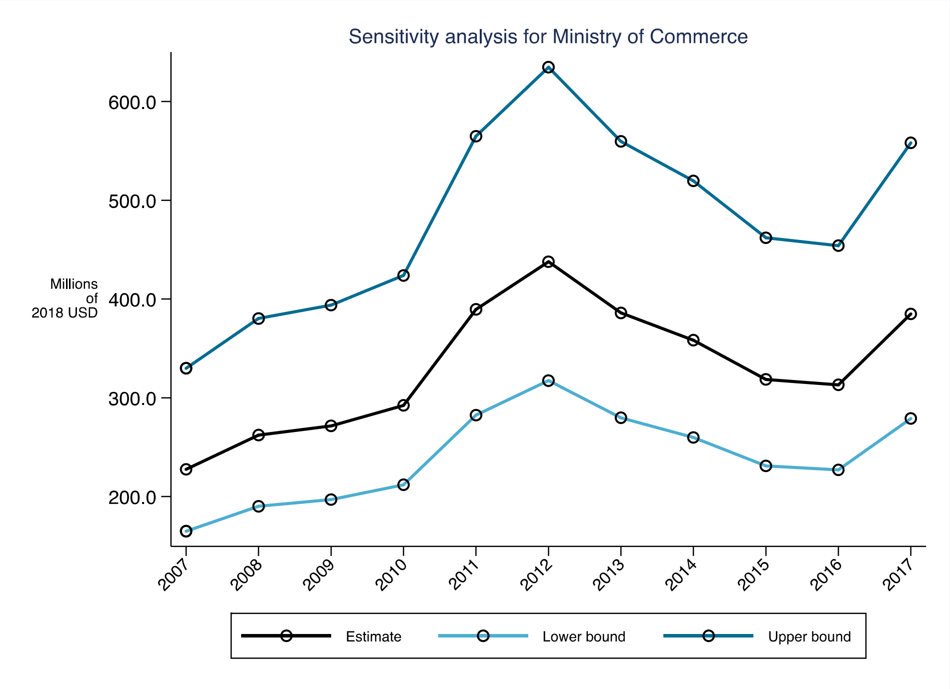

eFigure 8 Uncertainty level for China Ministry of Commerce scenario

We also assumed that 13.7% of the Ministry of Commerce's ODA is health and in the sensitivity analysis, we provided the lower bound and upper bound assuming that 10% and 20% of the ODA is health.

Scenario: Vary share of MOE scholarship cost by plus or minus 1 standard deviation and assume fixed proportion of students are medical students

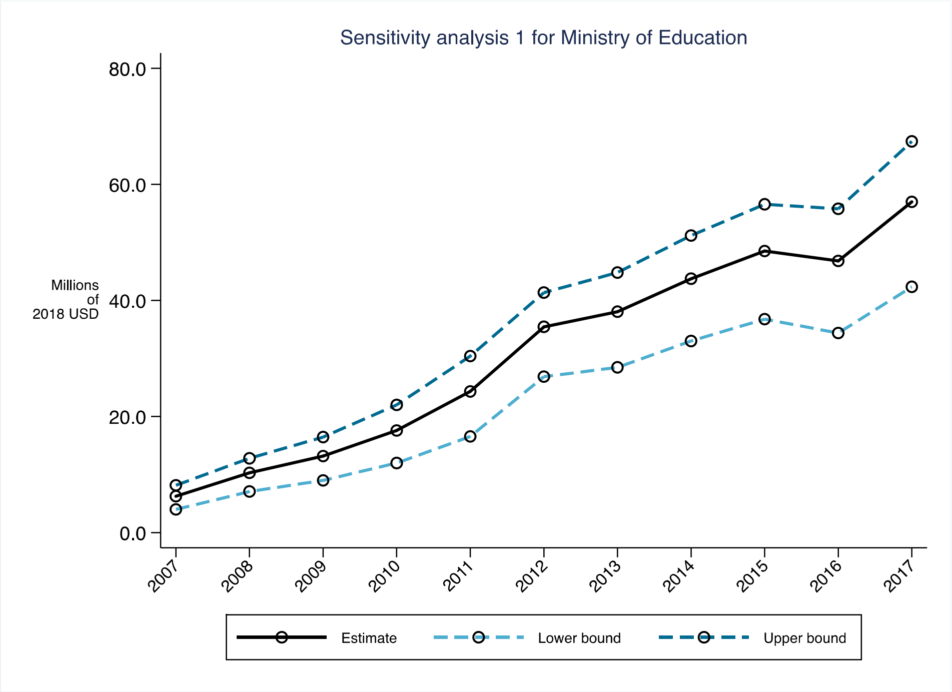

Scenario: Vary share of MOE student number by plus or minus 1 standard deviation and assume fixed proportion of scholarship cost

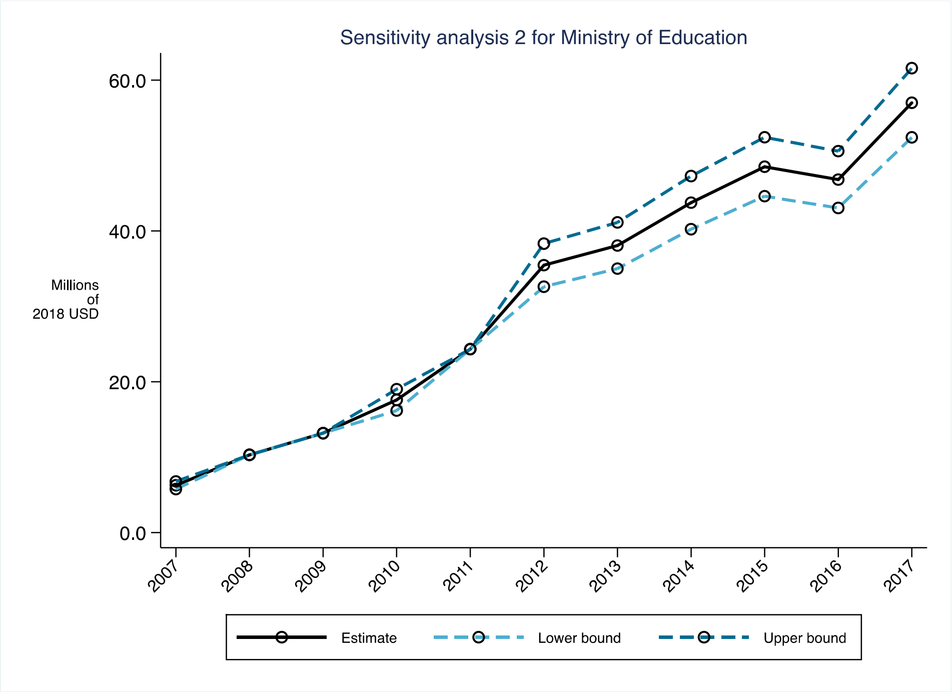

Scenario: Vary share of MOE scholarship cost by plus or minus 1 standard deviation and vary share of MOE student number by plus or minus 1 standard deviation

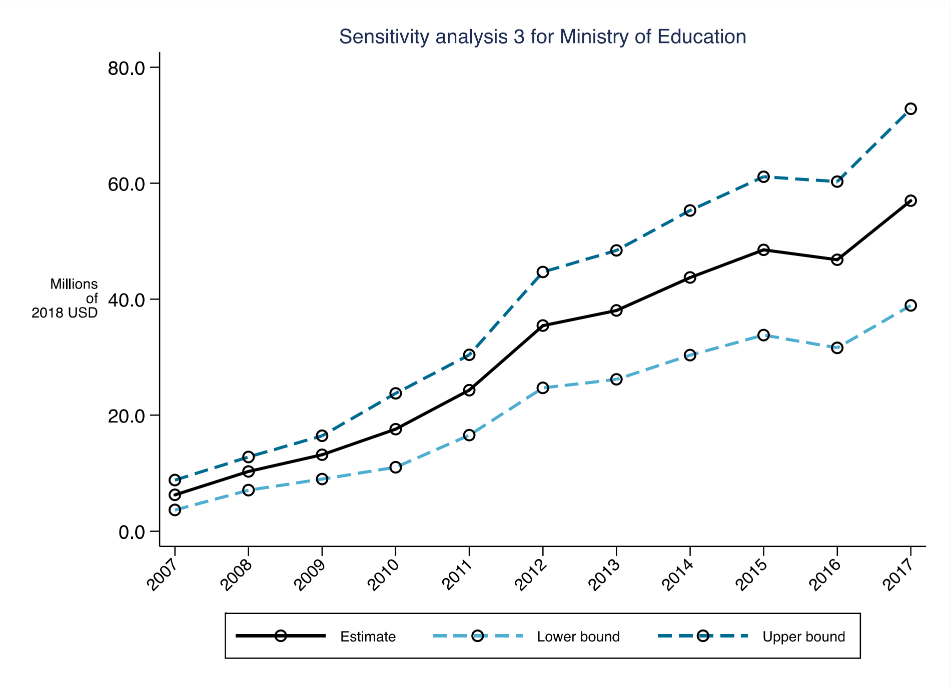

eFigure 9 Uncertainty level for China Ministry of Education scenario (a), (b) and (c)

Similarly, for the Ministry of Education, we provided our uncertainty level in 3 scenarios. In scenario (a), we estimated the range of cost per medical students through the standard deviation of the cost, with lower bound as 0.04 million of CNY in 2017 and upper bound as 0.06 million of CNY in 2017. In scenario (b), we estimated the number of medical students using the standard deviation of proportion of medical students among all students, and the range was 10.2%-12.0%. Finally, in scenario (c), we combined both scenario (a) and (b), and estimated the lowest bound and highest bound for Ministry of Education's DAH. The amount range was 38.93-72.82 million of 2018 USD in 2017.

Scenario: Vary share of EXIM ODA that is for health (1%-2%)

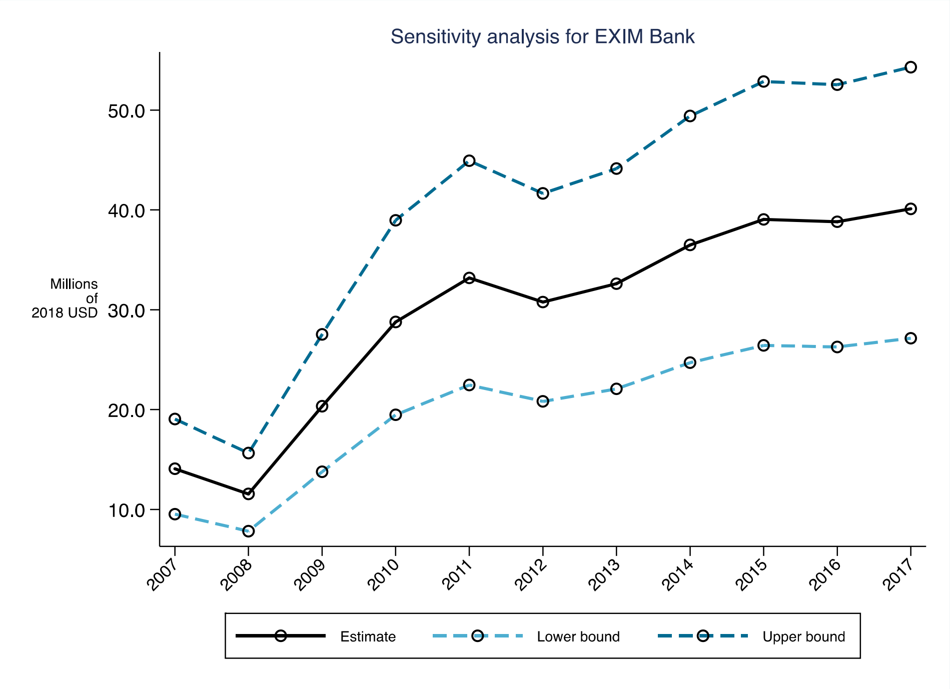

eFigure 10 Uncertainty level for China Export-Import Bank for the proportion of Export-Import Bank aid that is for health

For Export-Import Bank, our estimate assumed that 1.5% of EXIM's ODA are health projects, which we extracted from the AidData dataset. We provided the uncertainty level by estimating 1% and 2% of EXIM's ODA are health projects. As shown in eFigure 10, the dash lines indicated the modelled upper and lower bound of DAH provided through the EXIM bank. This agency shows the largest variation in range of possible estimates with the variation in assumptions.

Scenario: The combined lowest bound and highest bound across for all bilateral channels.

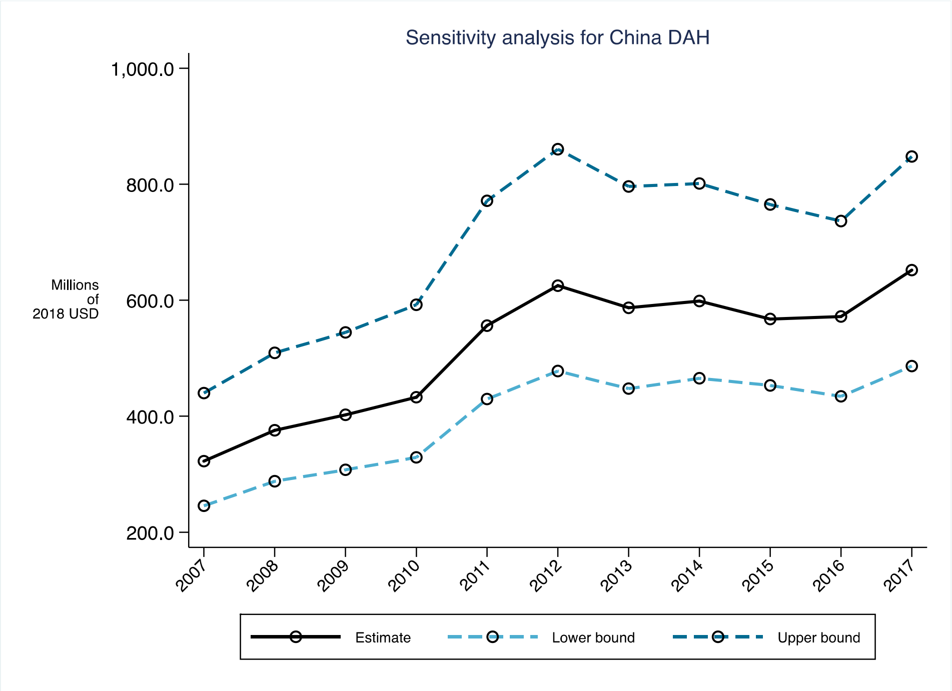

eFigure 11 Uncertainty level for China DAH

Finally, we considered all scenarios and combined the analysis to project the lowest bound and highest bound for China DAH. The result is shown in eFigure 11, in 2017 the lower bound was 486.4 million of 2018 USD and the higher bound was 847.7 million of 2018 USD.

Reference

1. The State Council. China's Foreign Aid (2011) [Internet]. [cited 2018 Aug 22]. Available from: http://english.gov.cn/archive/white_paper/2014/09/09/content_281474986284620.htm

2. The State Council. China's Foreign Aid (2014) [Internet]. [cited 2018 Aug 24]. Available from: http://english.gov.cn/archive/white_paper/2014/08/23/content_281474982986592.htm

3. Kitano N, Harada Y. Estimating China's Foreign Aid 2001–2013. J Int Dev. 2016 Oct 1;28(7):1050–74.

4. JICA Research Institute. A Note on Estimating China's Foreign Aid Using New Data: 2015 Preliminary Figures. [Internet]. 2018. Available from: https://www.jica.go.jp/jica-ri/publication/other/l75nbg000008yara-att/note_20170601.pdf

5. Shajalal M, Xu J, Jing J, King M, Zhang J, Wang P, et al. China's engagement with development assistance for health in Africa. Glob Health Res Policy [Internet]. 2017 Aug 9 [cited 2018 Sep 12];2. Available from: https://www.ncbi.nlm.nih.gov/pmc/articles/PMC5683463/

6. Liu P, Guo Y, Qian X, Tang S, Li Z, Chen L. China's distinctive engagement in global health. The Lancet. 2014 Aug;384(9945):793–804.

7. Bräutigam D. Aid 'With Chinese Characteristics': Chinese Foreign Aid and Development Finance Meet the OECD-DAC Aid Regime. J Int Dev. 2011;23(5):752–64.

8. Tang K, Li Z, Li W, Chen L. China's Silk Road and global health. The Lancet. 2017 Dec 9;390(10112):2595–601.

9. Brautigam D. The Dragon's Gift: The Real Story of China in Africa. Reprint edition. Oxford: Oxford University Press; 2011. 416 p.

10. Mckinsey & Company. Dance of the lions and dragons: How are Africa and China engaging, and how will the partnership evolve? [Internet]. 2017. Available from: www.mckinsey.com/africa-china

11. Yanzhong H. Domestic Politics and China's Health Aid to Africa. China Int J. 2014 Dec 15;12(3):176–98.

12. Dreher A, Fuchs A. Rogue Aid? The Determinants of China's Aid Allocation [Internet]. Rochester, NY: Social Science Research Network; 2011 Sep [cited 2018 Dec 18]. Report No.: ID 1926471. Available from: https://papers.ssrn.com/abstract=1926471

13. China Export-Import Bank. Introduction to Concessional Loan. [Internet]. Available from: http://www.chinca.org/cms/html/files/2013-12/16/20131216102948872930302.pdf

14. China Ministry of Commerce. Commerce Yearbook of China 2000-2016.

15. China Ministry of Commerce. China Ministry of Commerce Department Final Account 2007-2017.

16. China Ministry of Education. Education Yearbook of China 2000-2014. People's Education Press.;

17. China National Health Commission. Department of International Cooperation: Responsibility [Internet]. Available from: http://www.nhc.gov.cn/gjhzs/pzyzz/lists.shtml

18. China National Health Commission. China National Health Commission Department Final Account 2010-2017.

19. China International Development Cooperation Agency: Medical Aid [Internet]. [cited 2018 Nov 29]. Available from: http://www.cidca.gov.cn/ylyw.htm

20. All China Woman Federation. All China Woman Federation Website [Internet]. Available from: http://www.womenofchina.cn/

21. China-Africa Development Fund. China-Africa Development Fund Website [Internet]. Available from: http://www.cadfund.com/

22. Schickerling EJ. The role of the China Africa Development Fund in China's Africa policy [Internet] [Thesis]. Stellenbosch : Stellenbosch University; 2012 [cited 2018 Dec 18]. Available from: https://scholar.sun.ac.za:443/handle/10019.1/71761

23. China Development Bank: China-Africa Cooperation and Development Finance [Internet]. [cited 2018 Dec 18]. Available from: http://www.cdb.com.cn/xwzx/khdt/201809/t20180921_5421.html

24. China Ministry of Agriculture. China Ministry of Agriculture Website [Internet]. Available from: http://english.agri.gov.cn/

25. China Ministry of Civil Affairs. China Ministry of Civil Affairs Website [Internet]. Available from: http://english.gov.cn/state_council/2014/09/09/content_281474986284128.htm

26. China Ministry of Finance. China Ministry of Finance Department Budget [Internet]. 2017. Available from: http://m.mof.gov.cn/czsj/201804/P020180413300901890348.pdf

27. China Ministry of Human Resource and Social Security. China Ministry of Human Resource and Social Security Website [Internet]. Available from: http://www.mohrss.gov.cn/index.htm

28. China Ministry of Science and Technology. China Ministry of Science and Technology Website [Internet]. Available from: http://www.most.gov.cn/eng/

29. China National Development and Reform Commission. China National Development and Reform Commission Website [Internet]. Available from: http://en.ndrc.gov.cn/

30. China Foundation Center. China Foundation Center Website [Internet]. [cited 2018 Sep 13]. Available from: http://en.foundationcenter.org.cn/online.html

31. China Foundation for Poverty Alleviation. China Foundation for Poverty Alleviation Annual Report 2012-2017 [Internet]. Available from: http://www.cfpa.org.cn/information/institution.aspx

32. People's Bank of China. People's Bank of China Annual Report.

33. Red Cross Society of China. Red Cross Society of China Department Final Account 2013, 2016 [Internet]. Available from: http://www.redcross.org.cn/files/pdf/P020170721603559080590.pdf

34. Silk Road Fund. Silk Road Fund Website [Internet]. Available from: http://www.silkroadfund.com.cn/enweb/23773/index.html

35. China pledges 2 billion USD to support South-South cooperation - [Internet]. [cited 2018 Dec 18]. Available from: http://english.mofcom.gov.cn/article/zt_usvisit/news/201509/20150901125965.shtml

36. China State Oceanic Administration. China State Oceanic Administration Website [Internet]. Available from: http://english.gov.cn/state_council/2014/10/06/content_281474992889983.htm

37. China National Health Commission. China National Health Commission Department Budget 2015-2018.

38. China Ministry of Finance. Finance Yearbook of China 2000-2016. China Financial & Economic Publishing House.;

39. China Ministry of Commerce. China Ministry of Commerce Department Budget 2015-2018.

40. United Nations Office for the Coordination of Humanitarian Affairs (UNOCHA) Financial Tracking Service [Internet]. Available from: https://fts.unocha.org/data-search

41. China Ministry of Education. China Ministry of Education Department Final Account 2007-2017.

42. China Ministry of Education. Incoming international student statistics 2015 [Internet]. Available from: http://www.moe.gov.cn/jyb_xwfb/gzdt_gzdt/s5987/201604/t20160414_238263.html

43. China Ministry of Education. Incoming international student statistics 2016 [Internet]. Available from: http://www.moe.gov.cn/jyb_xwfb/xw_fbh/moe_2069/xwfbh_2017n/xwfb_170301/170301_sjtj/201703/t20170301_297677.html

44. China Ministry of Finance. Notice on Improving Incoming Foreign Scholarship Student's Stipend [Internet]. [cited 2018 Aug 23]. Available from: http://jkw.mof.gov.cn/zhengwuxinxi/zhengcefabu/201501/t20150121_1182625.html

45. People's Bank of China. Almanac of China's Finance and Banking 2005, 2006, 2007, 2008, 2010, 2011, 2012, 2013, 2014. Almanc of China's Finance and Banking Magazine Co. Ltd;

46. Dreher, Axel, Andreas Fuchs, Bradley Parks, Austin M. Strange, Michael J. Tierney. Aid, China, and Growth: Evidence from a New Global Development Finance Dataset. AidData Working Paper #46. Williamsburg, VA: AidData at William & Mary. [Internet]. [cited 2018 Sep 12]. Available from: https://www.aiddata.org/publications/aid-china-and-growth-evidence-from-a-new-global-development-finance-dataset

47. Development Assistance for Health Database 1990-2018 | GHDx [Internet]. [cited 2019 May 1]. Available from: http://ghdx.healthdata.org/record/ihme-data/development-assistance-health-database-1990-2018

48. Miyuki Parris. Project database 1990-2018 obtained through personal correspondence. Washington D.C.: The World Bank; 2019.

49. World Bank IDA. World Bank International Development Association (IDA) 18 Replenishment [Internet]. Available from: http://ida.worldbank.org/financing/replenishments/ida18-overview/ida18-replenishment

50. OECD. OECD Statistics [Internet]. [cited 2018 Dec 18]. Available from: https://stats.oecd.org/index.aspx?queryid=169

51. International Monetary Fund. World Economic Outlook Database April 2017 [Internet]. [cited 2018 Dec 18]. Available from: https://www.imf.org/external/pubs/ft/weo/2017/01/weodata/index.aspx

52. Chang AY, Cowling K, Micah AE, Chapin A, Chen CS, Ikilezi G, et al. Past, present, and future of global health financing: a review of development assistance, government, out-of-pocket, and other private spending on health for 195 countries, 1995–2050. The Lancet [Internet]. 2019 Apr 25 [cited 2019 May 4]; Available from: http://www.sciencedirect.com/science/article/pii/S0140673619308414

53. Institute for Health Metrics and Evaluation (IHME). Financing Global Health 2018: Countries and Programs in Transition. Seattle, WA: IHME, 2019. Available from: http://www.healthdata.org/policy-report/financing-global-health-2018-countries-and-programs-transition

54. OECD. Aid (ODA) by sector and donor [DAC5] [Internet]. Available from: https://stats.oecd.org/

55. OECD. Development Co-operation Report [Internet]. [cited 2019 May 6]. Available from: https://www.oecd-ilibrary.org/development/development-co-operation-report_20747721
